# A novel bispecific antibody platform to direct complement activity for efficient lysis of target cells

**DOI:** 10.1038/s41598-019-48461-1

**Published:** 2019-08-19

**Authors:** Jonathan W. Cruz, Ermelinda Damko, Bhavika Modi, Naxin Tu, Karoline Meagher, Vera Voronina, Hans Gartner, George Ehrlich, Ashique Rafique, Robert Babb, Priya Aneja, Terra B. Potocky, Amanda D’ Orvilliers, Alida Coppi, Sook Yen E, Haibo Qiu, Courtney M. Williams, Brandy L. Bennett, Gang Chen, Lynn Macdonald, William Olson, John C. Lin, Neil Stahl, Andrew J. Murphy, Christos A. Kyratsous, Brinda C. Prasad

**Affiliations:** Regeneron Pharmaceuticals Inc., Tarrytown, NY 10591 USA

**Keywords:** Biologics, Antimicrobials

## Abstract

Harnessing complement-mediated cytotoxicity by therapeutic antibodies has been limited because of dependency on size and density of antigen, structural constraints resulting from orientation of antibody binding, and blockade of complement activation by inhibitors expressed on target cells. We developed a modular bispecific antibody platform that directs the complement-initiating protein C1q to target cells, increases local complement deposition and induces cytotoxicity against target antigens with a wide-range of expression. The broad utility of this approach to eliminate both prokaryotic and eukaryotic cells was demonstrated by pairing a unique C1q-recruiting arm with multiple targeting arms specific for *Staphylococcus aureus*, *Pseudomonas aeruginosa*, B-cells and T-cells, indicating applicability for diverse indications ranging from infectious diseases to cancer. Generation of C1q humanized mice allowed for demonstration of the efficacy of this approach to clear disease-inducing cells *in vivo*. In summary, we present a novel, broadly applicable, and versatile therapeutic modality for targeted cell depletion.

## Introduction

Therapeutics that direct powerful effector functions of the immune system towards target cells hold great promise in settings of therapeutic resistance of infectious diseases and cancer. The complement system is an ancient and well-conserved branch of innate immunity that recognizes and clears pathogens by direct lysis^1,2^, opsonization^3^, and recruitment of effector cells^4,5^. Engaging these activities to clear pathogenic cells can provide a novel modality to add to the therapeutic arsenal.

Classical complement activation is initiated by the C1 complex, composed of C1q, C1r and C1s proteins. C1q is a hexameric protein comprised of one stalk domain and six head domains which recognize molecular patterns on cell surfaces, as well as antigen-antibody complexes via binding to IgG1 or IgG3 Fc domains. C1q binds to individual Fc domains with low affinity, but antigen-induced multimerization of Fc domains results in avidity-driven enhancement of C1q binding. Normally, C1q does not bind to healthy mammalian cells^6,7^ but opsonizes dying or abnormal cells and targets them for clearance^6^. Engagement of multiple C1q globular heads results in conformational changes and activation of the associated C1r and C1s proteases^7^. The ensuing serine protease cascade results in sequential recruitment and cleavage of complement components that culminates in the insertion of the lytic membrane attack complex (MAC, C5b9) pore causing rapid loss of cell viability. Cell-surface bound C1q can also be directly recognized by C1q binding proteins or a C1q receptor on a variety of effector cells leading to phagocytosis or cytotoxicity^8^. Taken together, C1q is an important component of the innate immune system.

Directed complement recruitment has not been effectively harnessed for broad therapeutic application. To date, therapeutic antibodies engaging complement do so via their Fc domains and therefore potency is dependent on the density and geometry of antibody binding to the target antigen. For example, anti-CD20 IgG1 mAbs, such as rituximab, induce complement-dependent cytotoxicity (CDC; i.e., MAC-mediated lysis) of CD20-expressing B cells *in vitro*, with potency related to CD20 density^9,10^. However *in vivo*, the relative contribution of CDC versus effector cell-mediated death such as antibody-dependent cell-mediated cytotoxicity (ADCC) is unknown. Efforts to engineer Fc domains with enhanced ability to recruit C1q and activate CDC, either by chimeric IgG1/IgG3 domains^11^ or by inducing Fc hexamerization through amino acid changes^9,12^, have been described. In contrast, directly recruiting C1q to cell surfaces using a C1q-binding domain has been less studied. In a previous study directing C1q to target cells, two single chain antibody fragments (scFvs) recognizing lysozyme and C1q were linked together to create a bispecific diabody. This diabody effectively lysed lysozyme-coated sheep erythrocytes by CDC^13^. However, to our knowledge, directed C1q deposition and lysis of target cells using bispecific antibodies (bsAbs) that recognize target cell specific proteins and recruit C1q have not been demonstrated.

While Gram-negative organisms are readily killed by complement, Gram-positive bacteria have been considered resistant due to their thick peptidoglycan cell wall, which is thought to prevent MAC insertion. Deposition of complement components, including the C5b9 complex, has been observed on the surface of Gram-positive organisms but cytotoxicity has not been detected^14–16^. Because of the relative refractiveness of the Gram-positive *S*. *aureus* to killing by complement, we chose this organism to test if recruiting complement can result in potent bactericidal activity against a normally resistant target. Furthermore, as the mechanism of complement killing is different from antibiotics, this approach is expected to have activity against antibiotic-resistant bacteria.

Here we introduce a C1q-recruiting bsAb, with one arm that binds C1q and the other arm that binds cell surface targets, that can enhance complement deposition and mediate cytotoxicity of bacterial and mammalian target cells. The bsAb was constructed based on the native human immunoglobulin format with a common light chain, purified by virtue of differential binding to protein A between the constant region of IgG1 and IgG3, as previously described^17^. We demonstrate that *S*. *aureus* is sensitive to the bactericidal activity of serum, and this activity is enhanced by the C1q-recruiting bsAb *in vitro* and *in vivo*. Importantly, we show that our C1q bispecific platform is broadly applicable and can mediate killing of bacterial and mammalian cells. Both Gram-positive and Gram-negative bacteria, tumor cell lines and immune cells, irrespective of target antigen chosen, can be lysed by recruiting the classical complement pathway. Taken together, we describe a platform for a novel complement-recruiting bispecific technology.

## Results

### Human serum kills *S*. *aureus* via terminal complement

**Figure 1:**
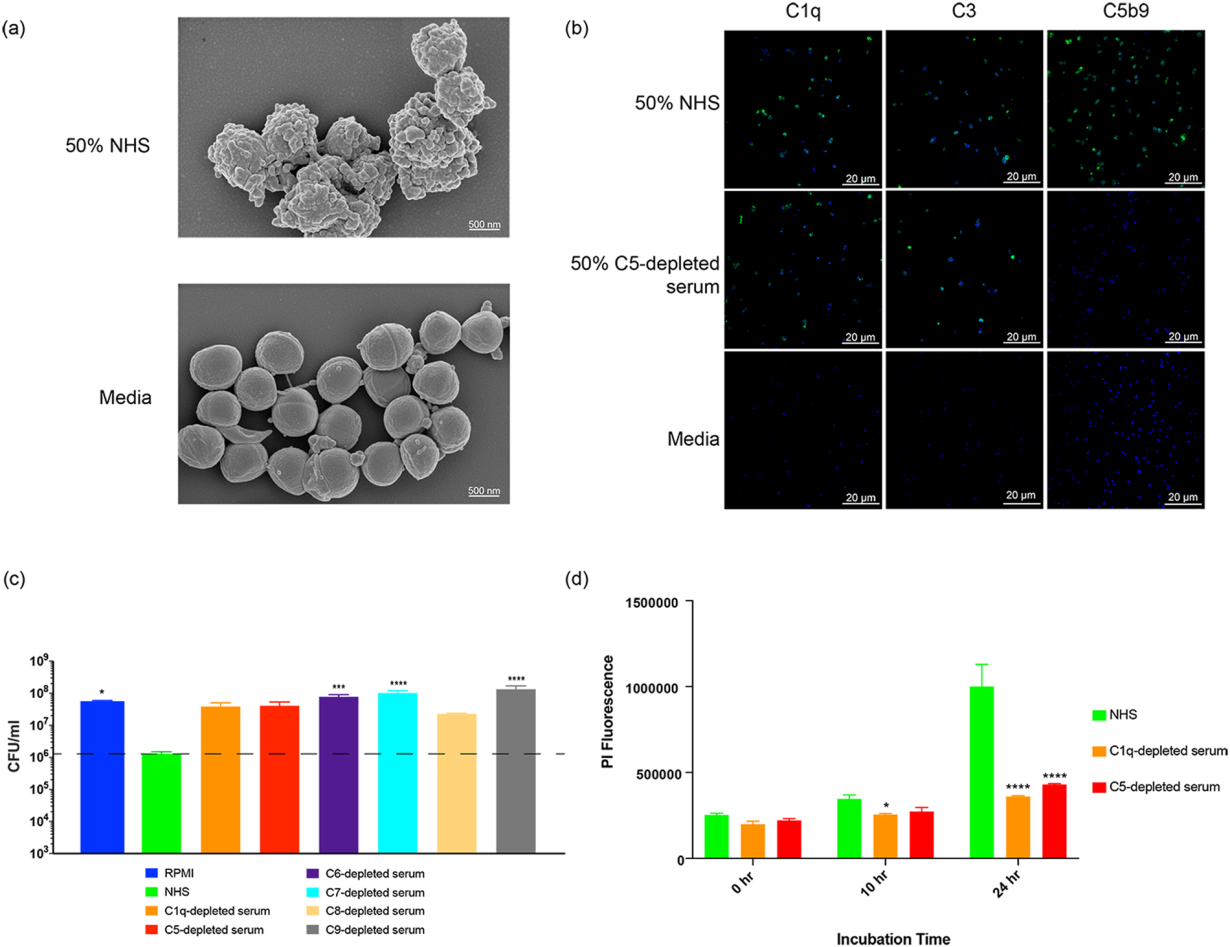
MAC formation and NHS-mediated reduction in the growth of *S*. *aureus* is dependent on a complete terminal complement pathway. (**a**) *S*. *aureus* was incubated with 50% NHS (top) or media (bottom) for 8 h and then visualized by scanning electron microscopy (SEM). (**b**) *S*. *aureus* was incubated with 50% NHS (top), 50% C5-depleted serum (middle) or media (bottom) for 1 h. Complement proteins indicated at the top of each column were detected using antibodies specific for C1q (far left), C3 (center) or C5b9 (far right) followed by an Alexa Fluor 488 conjugated secondary (green). Bacteria were stained with DAPI (blue) and then imaged using a Zeiss LSM780 confocal microscope. (**c**) The effect of human serum on growth of *S*. *aureus* was measured using serum killing assays. S. *aureus* was incubated with 50% of the indicated sera or medium for 24 h. After incubation, bacteria were enumerated by serial dilution and plating. Results are plotted as mean with standard deviation. *P < 0.05, ***P < 0.001, ****P < 0.0001, one-way ANOVA with Dunnett’s test showing significance compared to the NHS sample. (**d**) *S*. *aureus* was incubated with 50% NHS, 50% C1q-depleted serum or 50% C5-depleted serum for 0, 10, and 24 h at 37 °C. Uptake of the viability dye propidium iodide (PI) was assessed by measuring fluorescence (excitation 533 nm, emission 617 nm). Results are plotted as mean with standard deviation. *P < 0.05, ****P < 0.001, two-way ANOVA showing values significantly different than NHS.

Previous work has shown complement proteins, including C5b9 complexes, deposit on the surface of Gram-positive organisms after short incubations (1–2 h) in 10% normal human serum (NHS), however no functional consequence of deposition was observed^16,18,19^. To better understand how complement affects Gram-positive organisms, we first visualized *S*. *aureus* incubated with either 50% NHS (closer to physiological levels than previously tested) or media. Scanning electron microscopy (SEM) showed striking differences on the surface of *S*. *aureus* after 8 h of incubation with NHS (Fig. 1a). We confirmed complement deposition on *S*. *aureus* using immunofluorescence microscopy to visualize C1q, C3 and C5b9^20^ proteins. In the presence of 50% NHS, C1q, C3 and C5b9 were surface-bound and evenly distributed (Fig. 1b). As expected, depletion of C5 resulted in C1q and C3 deposition, but not C5b9.

To demonstrate the functional consequence of MAC formation, *S*. *aureus* were incubated with 50% NHS, NHS depleted of individual terminal complement components or media. After 24 h there was a 100-fold reduction in viable bacterial colonies compared to media, bactericidal activity was observed with NHS (Fig. 1c). Similar results were observed when ATP release was used to quantify viable bacteria (Supplementary Fig. 1). Growth in serum depleted of C1q or any terminal complement component (C5, C6, C7, C8, C9; Fig. 1c) was comparable to media. Furthermore, incubation of *S*. *aureus* with NHS, but not C1q or C5 depleted serum, resulted in uptake of the viability dye propidium iodide (PI; Fig. 1d). While PI staining at 0 h was similar in all test conditions, there was a significant increase in the amount of PI taken up in NHS treated samples after 24 h but not in complement depleted sera (Fig. 1d). Together, these results indicate that C1q-initiated complement activation and MAC formation results in *S*. *aureus* killing.

### A bispecific antibody directing C1q to *S*. *aureus* enhances complement deposition and results in cytotoxicity

**Figure 2:**
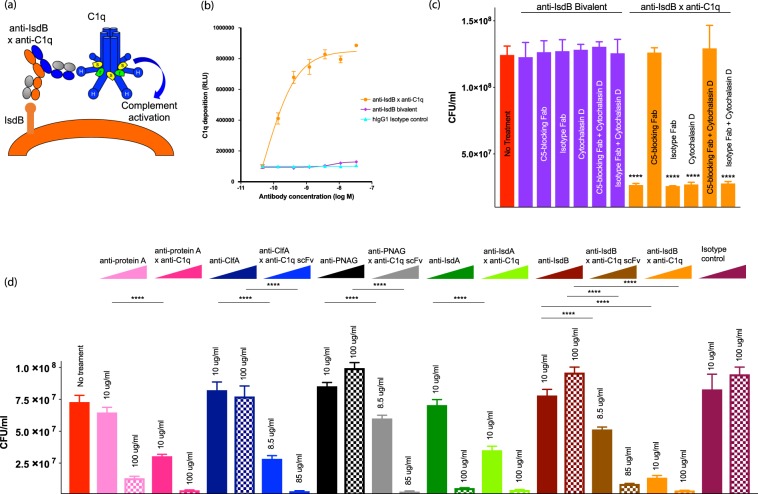
Bispecific antibodies targeting IsdB and C1q can increase complement deposition on the surface of *S*. *aureus* leading to cell death. (**a**) Proposed mechanism of action of the anti-IsdB x anti-C1q antibody. One arm of the antibody (orange and gray) targets the *S*. *aureus* surface antigen IsdB with high affinity. The other arm (blue and gray) targets the complement C1q protein. Engagement of multiple C1q heads by multiple bsAbs can lead to activation of complement. (**b**) C1q deposition on *S*. *aureus* was measured using a plate-based format. *S*. *aureus* Newman was coated onto plates and then incubated with NHS in the presence of anti-IsdB x anti-C1q bsAb, an anti-IsdB bivalent antibody or an isotype control antibody. C1q deposition was measured with a primary antibody specific for C1q and an HRP-conjugated secondary antibody. Results are plotted as mean ± standard deviation. (**c**) Whole blood assays were performed by incubating *S*. *aureus* Newman with blood alone (far left red column), blood with an anti-IsdB bivalent antibody (columns 2–7; purple) or blood with anti-IsdB x anti-C1q bsAb (columns 8–13; orange). The antibodies were combined with either a C5 blocking Fab (columns 3 and 9), an isotype control Fab (columns 4 and 10), or cytochalasin D (columns 5 and 11), both the C5-blocking antibody and cytochalasin D (columns 6 and 12) or both the isotype control Fab and cytochalasin D (columns 7 and 13). Bacterial growth was enumerated by plating. ****P < 0.0001, one-way ANOVA with Dunnett’s test showing samples significantly different from no treatment sample. Results are plotted as mean with standard deviation. (**d**) bsAbs recognizing C1q and different antigens on the surface of *S*. *aureus* were produced. Blood assays were performed as described above with two concentrations of antibody. Full length-antibodies were tested at 10 and 100 μg/ml, antibodies with an scFv arm were tested at 8.5 and 85 ug/ml (the molar equivalent of the full-length antibodies). Results are plotted as mean with standard deviation. *P < 0.05, **P < 0.01, ***P < 0.001, ****P < 0.0001, one-way ANOVA showing bsAb samples significantly different from the corresponding bivalent antibody.

After determining that complement can reduce *S*. *aureus* viability, we wanted to develop a strategy to increase both complement deposition and bacterial killing. We hypothesized that a C1q-recruiting bsAb could engage C1q, increase complement deposition and activate complement in a manner less dependent on antigen density and geometry of antibody binding. Engagement of multiple C1q globular heads is required for complement activation and MAC deposition, and multiple MAC pores are required to efficiently kill target cells^21–24^. To circumvent this, we designed a fully human IgG1 bsAb to recruit C1q directly to *S*. *aureus* (Fig. 2a). We selected the highly conserved and abundant bacterial surface expressed iron-regulated surface determinant protein B (IsdB) as the target. VelocImmune mice were immunized with purified human C1q and recombinant *S*. *aureus* IsdB to generate fully human antibodies against these two antigens^25,26^. We then paired IsdB binding arms with multiple C1q effector arms and chose the bsAb with maximal *S*. *aureus* killing activity in serum killing assays.

The binding properties of the anti-IsdB x anti-C1q bsAb were characterized by surface plasmon resonance (SPR)-Biacore. The bsAb and the parental, bivalent anti-IsdB antibody, when captured on the sensor chip surface, bound IsdB with nanomolar affinity (Table 1, Supplementary Fig. 2a). Biotinylated human C1q, captured on the chip at low density to mimic interaction in solution, bound to bsAb with a low K_D_ of 3.67 μM (Table 1, Supplementary Fig. 2b). To mimic the surface of *S*. *aureus*, IsdB was first captured on the chip surface, followed by injection of the bsAb and finally C1q, and the dissociation of C1q was measured. In this format, the affinity to C1q increased to 3.23 nM (Table 1, Supplementary Fig. 2c). The 1000-fold tighter binding results from avidity-driven interactions between the anti-C1q arm and multiple C1q heads.

**Table 1:** Binding parameters for anti-IsdB x anti-C1q.

Capture Surface	Test ligand	Biacore kinetic parameters for anti-IsdB x anti-C1q bispecific antibody binding to IsdB and C1q at 37 °C			
		k_a_ (M^−1^s^−1^)	k_d_ (s^−1^)	K_D_ (M)	T_1/2_ (min)
anti-IsdB x anti-C1q^a^	IsdB.6xHis	1.63 × 10^5^	5.57 × 10^−4^	3.43 × 10^−9^	21
anti-IsdB bivalent^a^	IsdB.6xHis	3.65 × 10^5^	6.40 × 10^−4^	1.75 × 10^−9^	18
Biotinylated hC1q^b^	anti-IsdB x anti-C1q	1.79 × 10^5^	8.71 × 10^−2^	4.78 × 10^−7^	0.13
IsdB.6xHis) + anti-IsdB x anti-C1q^c^	Human C1q	1.05 × 10^6^	5.13 × 10^−3^	4.90 × 10^−9^	2.3

Anti-IsdB x anti-C1q bsAb binds to C1q with low affinity and IsdB with high affinity. This bsAb was produced with one arm recruiting C1q and the other arm targeting the IsdB antigen present on the surface of *S*. *aureus*. The bsAb was characterized using Biacore.

^a^IsdB.6xHis was injected across an Fc-captured anti-IsdB x anti C1q chip surface.

^b^anti-IsdB x anti-C1q bsAb was injected across a streptavidin-captured biotinylated human C1q chip surface.

^c^anti-IsdB x anti-C1q bsAb and human C1q were sequentially injected across a chip surface of captured IsdB.6xHis protein.

We next demonstrated that anti-IsdB x anti-C1q dramatically increased recruitment of C1q to *S*. *aureus*. We measured C1q deposition on the surface of *S*. *aureus* Newman (a methicillin-sensitive strain, MSSA) resulting from binding of anti-IsdB x anti-C1q bsAb, the bivalent IsdB antibody or an isotype control antibody. While minimal C1q deposition was observed with the bivalent or isotype control antibody, incubation with anti-IsdB x anti-C1q bsAb resulted in robust, dose-dependent increase in C1q deposited on *S*. *aureus* (EC_50_ = 127 pM) (Fig. 2b, Supplementary Table 1).

The functional consequence of bsAb-mediated enhancement of C1q deposition was shown using a whole blood assay in which anti-IsdB x anti-C1q significantly inhibited *S*. *aureus* growth (Fig. 2c). While the bivalent IsdB antibody incubated with *S*. *aureus* Newman in human blood had no effect on bacterial growth after 24 h, anti-IsdB x anti-C1q bsAb reduced bacterial growth ~5-fold (Fig. 2c). This activity was dependent on complement activity: blocking phagocytosis with cytochalasin D had no effect on anti-IsdB x anti-C1q activity, whereas C5 blockade completely abrogated bsAb cytotoxicity (Fig. 2c). As a corollary test, we used flow cytometry to measure C3b deposition on *S*. *aureus* with NHS and NHS pre-adsorbed to *S*. *aureus* as a source of complement (Supplementary Fig. 3). C3b deposition was unchanged in the presence of the anti-IsdB x anti-C1q bsAb compared to control bsAb, a control bivalent antibody and no antibody. Additionally, to address if the anti-IsdB x anti-C1q bsAb activity is reliant on C1q recruitment through the Fab or Fc portions of this antibody, we tested an IgG4 version of the molecule. The IgG4 Fc has been demonstrated to bind less efficiently to C1q^27^. Killing was similar with both the IgG1 and IgG4 Fc, demonstrating that activity can be attributed to the C1q recruitment via the Fab arm of the bsAb (Supplementary Fig. 4a).

We performed several control experiments to assess if the anti-C1q effector arm can activate complement in the absence of target. To demonstrate the dependence on the target for bsAb activity, we used two control antibodies which recognize non-*Staphylococcal* targets, CD20 or EGFRvIII, paired with the same C1q effector arm (Supplementary Fig. 4b). *S*. *aureus* survival in the presence of the two control antibodies was similar to survival in untreated blood. Next, we added the anti-IsdB bivalent antibody, anti-IsdB x anti-C1q and anti-EGFRvIII x anti-C1q bsAbs to NHS and measured complement components by Luminex to assess complement activation and consumption (Supplementary Fig. 5). Concentrations of complement components and breakdown products (C1q, C4, C4b, C2, C3, C3b, C5, and C5a) after 1 h incubation at 37 °C were similar across all treatments. Finally, we used a monocytic cell line (U937) expressing high- and low-affinity Fcɣ receptors^28^ to perform a CDC-assay and examine potential side-effects of complement activation as a result of Fc-dependent bsAb binding. At antibody concentrations as high as 100 nM we did not see any cytotoxicity (Supplementary Fig. 6a,b).

To assess the role of antigen density on the C1q-recruiting bsAb activity, the same C1q-recruiting arm was paired with antibody arms targeting Protein A, clumping factor A (ClfA), poly-N-acetylglucosamine (PNAG) and iron-regulated surface determinant protein A (IsdA). Each of these antibodies bound C1q and *S*. *aureus* with high affinity (Supplementary Tables 1 and 2), and increased *S*. *aureus* killing compared to the corresponding bivalent antibody (Fig. 2d). The similar potency of these bsAbs, despite the variability in surface densities of the *S*. *aureus* target antigens (Supplementary Table 3), indicates that enhancing local concentrations of C1q on the cell surface overcomes the low efficiency of complement-mediated cytotoxicity. These results demonstrate that anti-IsdB x anti-C1q enhances killing of *S*. *aureus* and supports a C1q-recruiting bsAb approach to augment complement activity.

### Anti-IsdB x anti-C1q improves clearance of *S*. *aureus* in C1q-humanized mice

**Figure 3:**
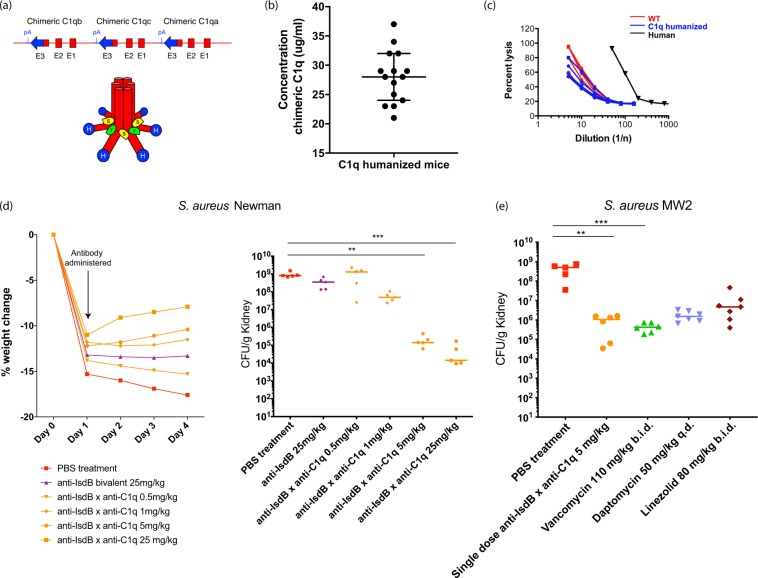
The IsdB x C1q bsAb increases clearance of *S*. *aureus* in mice with humanized C1q heads. (**a**) C1q humanized mice were produced by knocking out the mouse *C1qA*, *C1qB* and *C1qC* genes. They were replaced with chimeric versions of the genes in which the N-terminus of the gene encoding the collagen-like tail was mouse, but the C-terminus of the gene encoding the globular head was human. The resulting locus (not to scale) is shown with human sequences in blue and mouse sequences in red. C1q exons are labeled E1, E2, and E3. pA, polyA sequence (top). A representation of the resulting C1 complex is shown below. Human C1q heads are shown in blue, mouse C1q stalks are shown in red, mouse C1r is shown in green and mouse C1s is shown in yellow. (**b**) Quantitative measurement of the expression of chimeric C1q peptides was assessed via LC-MS/MS. Serum was isolated from individual C1q humanized mice and the concentration of humanized mouse C1q specific peptides was determined. Results are plotted as median and interquartile range. (**c**) CH50 hemolysis assays were used to determine the activity of serum from humanized C1q mice. Sheep erythrocytes were sensitized to complement lysis by incubation with rabbit anti-sheep antibodies. Dilutions of wild-type and C1q humanized mouse serum, as well as human serum, were then added. Lysis of the red blood cells was used to determine the activity of the sera. (**d**) C1q humanized mice were infected with 1.5 × 10^8^ CFU *S*. *aureus* Newman (MSSA strain). One day after infection, mice were treated with a single dose of the indicated antibody. Weights were recorded each day for four days and percent weight change compared to day 0 is shown (left). Mean weight change for each group of mice is plotted. On day 4, kidneys were harvested, dissociated, and serially diluted. Organ burden was determined by plating (right). The organ burden of each mouse is indicated along with the median value for each group. (**e**) C1q humanized mice were infected with 1.3 × 10^8^ CFU *S*. *aureus* MW2 (MRSA strain) on day 1. One day after infection they were treated with either PBS (no treatment), a single dose of anti-IsdB x anti-C1q antibody, 110 mg/kg vancomycin twice daily, 50 mg/kg daptomycin once daily or 80 mg/kg linezolid twice daily. On day 4, kidneys were harvested, dissociated, and serially diluted. Organ burden was determined by plating. The organ burden of each mouse is indicated along with the median value for each group. (**d**,**e**) **P < 0.01, ***P < 0.001, nonparametric Kruskal-Wallis ANOVA with Dunn’s test showing values significantly different from the no treatment samples.

After showing *in vitro* efficacy of anti-IsdB x anti-C1q bsAb, we set out to determine if enhanced cytotoxicity could be observed *in vivo*. Because the anti-C1q arm of the bsAb is specific for human C1q, and does not bind mouse C1q, we generated mice that encode the human sequence of the globular head of C1q to enable *in vivo* efficacy models. Mouse *C1qA*, *C1qB* and *C1qC* genes were replaced with chimeric versions, in which the N-terminus of the gene encoding the collagen-like tail was mouse sequence, and the C-terminus of the gene encoding the globular head was human (Fig. 3a, Supplementary Fig. 7a–c, Supplementary Tables 4 and 5). This resulted in a C1q protein that could bind the bsAb while still interacting with the rest of the mouse complement pathway. The genes remained under the control of the native mouse C1q promoter and expression of the chimeric proteins in serum was confirmed by mass spectrometry. On average these mice expressed 28 µg/ml of chimeric C1q with values ranging from 21 to 37 µg/ml (Fig. 3b). While C1q levels in the humanized mice were lower than in humans (average C1q level ~100 µg/ml^29^) or wild-type mice (average C1q level in C57Bl/6 J ~110 µg/ml^30^), the chimeric C1q was functional in an antibody-opsonized sheep erythrocyte hemolysis assay. The C1q-humanized mouse serum was as active as wild-type mouse serum (Fig. 3c) indicating that the chimeric C1q protein interacts normally with the mouse complement system.

Using these C1q humanized mice, we demonstrated that a single dose of anti-IsdB x anti-C1q bsAb significantly improved clearance of *S*. *aureus in vivo*. We first established a *S*. *aureus* systemic infection model: mice infected intraperitoneally (IP) with *S*. *aureus* exhibit dramatic weight loss and extensive bacterial dissemination to organs. *S*. *aureus* Newman infected mice were treated with PBS, the bivalent anti-IsdB antibody or anti-IsdB x anti-C1q on Day 1 (Fig. 3d and Supplementary Fig. 8b–e). One day after infection, the mice in each group lost 10–15% of their body weight. In PBS treated animals, weight loss continued after day 1, whereas the weight of mice stabilized by day 1 in mice treated with 25 mg/kg bivalent anti-IsdB antibody (Fig. 3d left). Remarkably, treatment with as little as 1 mg/kg anti-IsdB x anti-C1q stabilized weight after day 1, and doses of 5 and 25 mg/kg resulted in weight gains suggesting rapid reduction in bacterial load (Fig. 3d left). Mice treated with PBS and anti-IsdB bivalent antibody had 10^8^ bacteria per gram in their kidneys on Day 4 (Fig. 3d right). Conversely, treatment with as little as 1 mg/kg anti-IsdB x anti-C1q reduced kidney bacterial burden by at least 1-log, with a 4-log reduction observed at the 5 mg/kg dose (Fig. 3d right). Comparable reductions in bacterial burdens were observed in the hearts, livers, lungs and spleens (Supplementary Fig. 8b–e; respectively). Furthermore, similar trends in body weight and bacterial organ burden were observed with a methicillin resistant (MRSA) strain, *S*. *aureus* CA-127 (Supplementary Fig. 8a–e). These results demonstrate that while a bivalent anti-IsdB IgG1 antibody does not reduce *S*. *aureus* bacterial burden *in vivo*, the anti-IsdB x anti-C1q bsAb significantly enhances bacterial clearance.

We next showed that a single dose of anti-IsdB x anti-C1q is as effective at reducing *S*. *aureus* burden as daily dosing of the standard of care antibiotics vancomycin, linezolid and daptomycin. Mice were infected with *S*. *aureus* MW2, a MRSA strain. On Day 1, mice were treated with either PBS, 5 mg/kg anti-IsdB x anti-C1q bsAb, or standard of care antibiotics: 110 mg/kg vancomycin twice daily (b.i.d.), 50 mg/kg daptomycin once daily or 80 mg/kg linezolid b.i.d.^31^. While treatment with all antibiotics resulted in lower bacterial burden compared to PBS mice (daptomycin ~330-fold reduction; linezolid ~100-fold reduction), treatment with anti-IsdB x anti-C1q bsAb or vancomycin had significantly lower burden of *S*. *aureus* in their kidneys, with greater than 500-fold reduction (Fig. 3e). A single dose of the bsAb was as effective in clearing bacteria as daily vancomycin treatment.

### The C1q-recruiting bispecific platform is broadly applicable for directed cytotoxicity against multiple cell types

**Figure 4:**
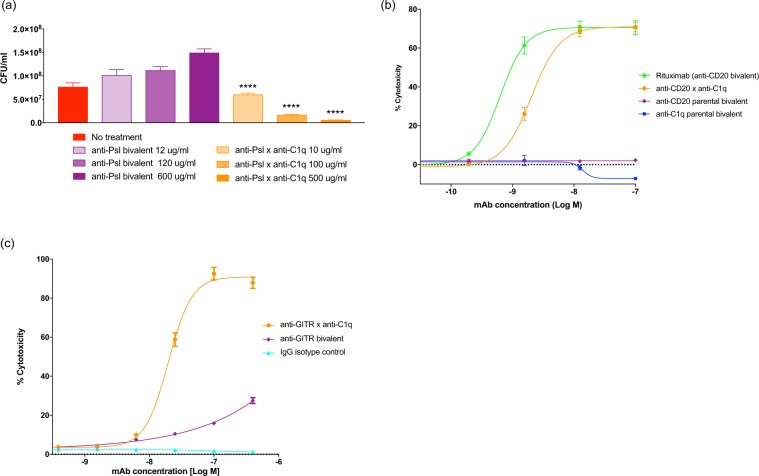
C1q-recruiting bsAbs bind to target cells and mediate cytotoxicity. (**a**) Growth of *P*. *aeruginosa* PAO1 in human blood was determined in the presence of the indicated antibodies and in blood or media (TSB) alone. ****P < 0.0001, two-way ANOVA showing bsAb samples significantly different from the corresponding bivalent antibody. Results are plotted as mean with standard deviation. (**b**,**c**) The ability of antibodies to mediate target cell lysis was measured using CDC assays. Increasing concentrations of the indicated antibodies were incubated with 5% NHS and Raji cells (**b**; human B-cell line) or Jurkat cells expressing human GITR and CD20 (**c**; human T-**c**ell line) for 2 or 3.5 h respectively. Cell lysis was quantified using the CytoTox-Glo assay which measures the release of proteases from dead cells. Percent cytotoxicity was calculated as the background subtracted experimental signal divided by the background subtracted maximum lysis signal. Results are plotted as mean ± standard deviation.

Having demonstrated efficacy of a C1q-recruiting bsAb against *S*. *aureus*, we investigated whether C1q-mediated activity could be used to effectively lyse Gram-negative bacteria and malignant mammalian cells with vastly different cell surfaces. We paired the C1q-recruiting effector arm with surface antigen targeting arms on three diverse cell types: Psl on Gram-negative *Pseudomonas aeruginosa*, CD20 on B-cells and glucocorticoid-induced TNFR-related protein (GITR) on T-cells. We found that each bsAb bound to both C1q (Supplementary Table 6) and their respective target cell (Table 2, Supplementary Table 7, and Supplementary Fig. 9) and further showed that each bsAb induced CDC of their target cell in the presence of complement (Fig. 4).

**Table 2:** Binding characteristics of parental bivalent and C1q-targeting bispecific antibodies.

Antibody	Cell type	Maximum binding (RLUs)	EC_50_ (M)
anti-Psl x anti-C1q	*Pseudomonas aeruginosa* PAO1	3.90 × 10^5^	4.32 × 10^−9^
anti-Psl bivalent	*Pseudomonas aeruginosa* PAO1	4.77 × 10^5^	3.45 × 10^−10^
anti-CD20 x anti-C1q	Raji- human B-cell	1.64 × 10^6^	3.57 × 10^−8^
anti-CD20 parental bivalent	Raji- human B-cell	1.71 × 10^6^	6.03 × 10^−9^
anti-CD20 bivalent, Rituximab	Raji- human B-cell	1.85 × 10^6^	3.14 × 10^−9^
anti-C1q parental bivalent	Raji- human B-cell	No binding	No binding
anti-GITR × anti-C1q	Jurkat/hGITR/hDC20- human T-cell	7.67 × 10^5^	3.10 × 10^−8^
anti-GITR bivalent	Jurkat/hGITR/hDC20- human T-cell	8.97 × 10^5^	3.07 × 10^−9^
anti-FelD1 isotype control	Jurkat/hGITR/hDC20- human T-cell	No binding	No binding

C1q-recruiting bsAbs bind to target cells with high affinity. Binding of antibodies to target cells was determined using ELISA (anti-Psl x anti-C1q and anti-CD20 x anti-C1q) or flow cytometry (anti-GITR x anti-C1q). Shown are maximum binding values for the antibody obtained in each assay and EC_50_ values for binding.

*P*. *aeruginosa*, an opportunistic human pathogen, is a significant source of disease in hospitals and among immunocompromised individuals due to antibiotic resistance^32^ and biofilm formation mediated by its surface exopolysaccharides^33^. We paired the anti-C1q effector arm with a targeting arm to the abundant exopolysaccharide, Psl. Both the anti-Psl x anti-C1q bsAb and the anti-Psl bivalent antibody bound to *P*. *aeruginosa* (EC_50_ 4.32 nM vs. 0.35 nM, respectively; Table 2). Addition of anti-Psl x anti-C1q bsAb to *P*. *aeruginosa* in human blood resulted in over a 1-log reduction in bacteria after 24 h compared to untreated control or anti-Psl bivalent antibody (Fig. 4a). This result demonstrated that the C1q-recruiting bsAb platform is effective against both Gram-positive and Gram-negative bacteria and further showed that adding a C1q-recruiting arm to an antibody that lacks intrinsic CDC activity can result in a molecule that leads to significant complement activation.

We next investigated the effects of the C1q-recruiting bsAbs against different lymphocytes that have been implicated in human disease pathogenesis. Rituximab, an anti-CD20 antibody, is the benchmark therapeutic used to treat B-cell lymphomas and induces complement-dependent lysis of lymphoma cells^34,35^. To evaluate whether the C1q platform is capable of conferring CDC activity to an otherwise CDC-incompetent antibody, we chose a clone of an anti-CD20 antibody that had no measurable CDC activity in the bivalent wild-type IgG1 format and paired it with the C1q effector arm. Consistent with its monovalent nature of CD20 binding, the anti-CD20 x anti-C1q bsAb bound CD20 expressing B-cells (Raji cell line) less tightly than either the parental anti-CD20 or the anti-CD20 antibody Rituximab (EC_50_ 35.7 nM vs. 6.03 nM vs. 3.14 nM, respectively; Table 2). Nevertheless, in the CDC assay the anti-CD20 x anti-C1q bsAb demonstrated an EC_50_ of 2.4 nM and 70% maximum cytotoxicity against Raji (Fig. 4b), a significant increase compared to the parental bivalent anti-CD20 antibody. Similar results were obtained using 50% NHS (Supplementary Fig. 10a).

T-regulatory cells may contribute to infection, autoimmunity, and cancer^36^. GITR, a TNF superfamily receptor, is highly expressed on tumor-infiltrating regulatory T-cells and has been used as a target to deplete regulatory T-cells in the tumor by ADCC to enhance the efficacy of cancer immunotherapy^37^. We asked whether it is possible to deplete GITR^+^ T-cells with a predominantly CDC mechanism by employing the bsAb format with a C1q-effector arm. We selected an anti-GITR bivalent antibody that had limited CDC activity against a Jurkat T-cell line engineered to overexpress GITR and paired the anti-GITR arm with the anti-C1q arm with the expectation of boosting the lytic activity. The bsAb efficiently bound Jurkat cells (EC_50_ of 31.0 nM compared to 3.07 nM for the bivalent; Table 2). Despite the increased expression levels of complement inhibitors on the surface of Jurkat cells compared to Raji cells (Supplementary Table 8), the anti-GITR x anti-C1q bsAb had significant CDC activity (EC_50_ 20 nM) with nearly 90% cytotoxicity (Fig. 4c), a 3-fold increase in maximum killing compared to the bivalent antibody. Similar results were obtained using 50% NHS (Supplementary Fig. 10b). These results demonstrate that a C1q-recruiting platform can result in potent, targeted killing of diverse cell types by either imparting novel CDC activity or enhancing pre-existing CDC activity of a given antibody.

## Discussion

To harness the potent killing activity of the complement system for therapeutic potential, we developed a fully human bispecific antibody platform that recruits C1q to lyse disease-causing cells. To demonstrate the utility of this platform, we initially chose *S*. *aureus*, an organism widely thought to be resistant to the cytotoxic effect of MAC. Using the C1q-directed bispecific approach, we enhanced complement deposition and killing *in vitro* and *in vivo*. Depletion of C5, a terminal complement component, abrogated the effect, demonstrating a dependence on MAC-mediated lysis. We further showed that this approach works using other *S*. *aureus* targets with different densities. Lastly, we extended these results to multiple cell types – including *P*. *aeruginosa*, Raji cells and Jurkat cells – to demonstrate the general applicability of this therapeutic approach.

Our study shows that Gram-positive bacteria are susceptible to direct complement-mediated lysis. MAC was not thought to play a role in control of Gram-positive infections in humans since direct killing had not been observed^16,19^, and both terminal complement deficiencies and blockade of C5 with eculizumab result only in increased susceptibility to Gram-negative bacteria of the genus *Neisseria*^38^. However, *S*. *aureus* do possess terminal complement inhibitors such as SSL7^39,40^ indicating a role for blocking MAC activity, and humans with deficiencies in early complement components (e.g., C1q, C2, C3, C4) are susceptible to multiple bacterial infections including *S*. *aureus*^41^. Therefore, the efficacy of our bsAb suggests that increasing bacterial cell surface localization of complement components, such as C1q, can be harnessed to kill Gram-positive bacteria through the formation of MAC.

Our study illustrates the wide-applicability of the C1q-bispecific platform approach by demonstrating targeted killing of both bacterial and mammalian cells. Additionally, this approach reduces the dependence on the antigen density and orientation of antibody binding to antigen in order to engage multiple C1q heads. The C1q-recruiting bsAb can activate complement when targeted to many different antigens (Figs 2d and 4a–c) including multiple *S*. *aureus* antigens, as well as antigens on *P*. *aeruginosa*, B-cells and T-cells. The platform should also be widely applicable to patients of different ages, genders, and immune status: the classical complement pathway and its protein components are abundant in circulation and stably expressed^42^, whereas immune effector cells such as neutrophils, macrophages and T-cells have impaired function with age^43–45^. Therefore, a C1q-based therapeutic has the potential to be used in immune compromised patients including those on immunosuppressive therapies. Furthermore, the platform may have the potential to be combined with existing treatments, such as antibiotics and tumor targeting agents.

While the terminal complement pathway is a potent mediator of cytotoxicity, there may be limitations to this approach. Although abundant, the amount of complement present in circulation is finite and may be transiently consumed as a result of bsAb treatment. However, localized production of complement components in disease settings may remain high (e.g., through the production of C1q by macrophages^46^), and further investigation is needed to determine if this affects the activity of the bsAb. Another possible limitation is the ability of bacteria and mammalian cells to escape lysis by expressing membrane complement inhibitors that block completion of the terminal complement pathway by sequestering complement components. Based on differential expression of these inhibitors, cell types have varying susceptibilities to complement mediated killing. Therefore, the threshold of complement deposition needed to achieve killing is different between cell types. Increasing complement deposition on the surface of cells with the bsAb has the potential to overcome inhibitor mediated resistance to killing. This is supported by the increase in cytotoxicity against both Raji and Jurkat cells (Fig. 4b,c) despite the intrinsic expression of varying levels of surface complement inhibitors (Supplementary Table 8). Additional studies will help us determine the full breadth of the therapeutic potential of this platform.

Immunomodulatory therapeutics, including bsAbs, are currently being investigated as powerful ways to eliminate a variety of disease-causing cell types. The C1q-recruiting platform leverages the cytotoxic effector functions of the innate immune system. The bsAbs we developed increased complement-mediated killing of bacterial and mammalian cells when targeted to antigens with diverse characteristics, despite the presence of complement inhibitors expressed by the target cells. The C1q-recruiting platform described here promises to open up new avenues of therapeutic development for overcoming resistance to existing therapies in infectious and other human diseases.

## Methods

### Bacterial strains and growth conditions

*S*. *aureus* Newman wild-type (wt) and *S*. *aureus* Newman Δ*spa* (an isogenic *S*. *aureus* in which the antibody binding protein, protein A, has been deleted) were kindly provided by Dr. T. J. Foster. *P*. *aeruginosa* PAO1 was obtained from ATCC. *S*. *aureus* strains were grown in phenol-free RPMI (Gibco) at 37 °C unless otherwise noted. *P*. *aeruginosa* was grown in LB (Teknova) at 37 °C unless otherwise noted.

### Scanning electron microscopy (SEM) visualization of *S*. *aureus*

*S*. *aureus* Newman Δ*spa* was grown in phenol-free RPMI medium (Gibco) at 37 °C overnight. The bacteria were washed two times with PBS and resuspended in 0.05% bovine serum albumin (BSA) in phenol-free RPMI to an OD_600_ ~ 1. 500 μl of bacteria was mixed with 500 μl of NHS (BioIVT) or 500 μl 0.05% BSA in phenol-free RPMI. The samples were incubated at 37 °C for 8 h with shaking. The bacteria were then washed three times with PBS. For EM analyses, samples were fixed 1:1 with a 2x fixative [5% glutaraldehyde, 4% paraformaldehyde (PFA) in 0.2 M sodium cacodylate buffer] and dehydrated through a graded series of ethanol. Samples were critical point dried using liquid carbon dioxide in a Tousimis Samdri 795 Critical Point Drier, coated with carbon in a Quorum EMS 150T ES (Quorum Technologies Ltd) and examined in a Zeiss Supra Field Emission Scanning Electron Microscope (Carl Zeiss Microscopy), using an accelerating voltage of 8 KV.

### Determination of complement deposition by fluorescent microscopy

*S*. *aureus* Newman Δ*spa* was grown in phenol-free RPMI medium (Gibco) at 37 °C overnight. The bacteria were washed once with PBS and resuspended in 3% BSA in PBS. They were blocked for 30 min at room temperature, washed twice with PBS and resuspended in 0.05% BSA in phenol-free RPMI to an OD_600_ ~ 1. 500 μl of bacteria was mixed with 500 μl of 20% NHS (BioIVT), 500 μl 20% C6-depleted serum (Quidel) or 500 μl 0.05% BSA in phenol-free RPMI. The samples were incubated for 1 h at 37 °C with shaking, then the bacteria were washed three times with PBS and resuspended in 1 ml of 1% BSA in PBS. Samples were aliquoted and incubated with goat anti-human C1q (Genway Biotech, Inc.), mouse anti-human C3 (Biolegend) or mouse anti-human C5b9 (Abcam) (at a final concentration of 1 μg/ml) for 1 h at room temperature and then washed three times with PBS. There were resuspended in 100 μl of 1% BSA in PBS and then probed with donkey anti-goat IgG conjugated to Alexa Fluor 488 (1:4000; ThermoFisher). Mouse IgG1 and mouse IgG2a antibodies (produced at Regeneron) against non-complement antigens were used as controls. DAPI (4′,6-diamidino-2-phenylindole, dihydrochloride, Biolegend) was added to samples at 5 μg/ml and they were incubated for 45 min at room temperature. The samples were then visualized on a Zeiss LSM780 confocal microscope.

### Serum killing assay

*S*. *aureus* Newman was grown in phenol-free RPMI medium (Gibco) at 37 °C overnight. The bacteria were washed one time with PBS and resuspended in 0.05% BSA in RPMI to an OD_600_ ~ 0.5 and further diluted 1:1000 to a final concentration of ~1.0 × 10^5^ CFU/ml. Bacteria were subsequently mixed with an equal volume of NHS (Quidel) or human serum depleted of a terminal complement protein (Quidel) such that the final concentration of serum was 50%. The samples were incubated at 37 °C for 24 h with shaking. Surviving bacteria were serially diluted in PBS and plated onto LB agar plates for enumeration of CFUs. Bacterial survival was also measured using BacTiter-Glo (Promega) according the manufacturer’s instructions. Briefly BacTiter-Glo buffer was added to BacTiter-Glo substrate and then 100 μl of this reagent was added to 100 μl of bacterial sample in a 96-well plate. The plate was mixed for 30 s on an orbital plate shaker (Eppendorf) and then incubated for 5 min at room temperature. Luminescence was detected using a SpectraMax i3x plate reader (Molecular Devices) and results are plotted as mean with standard deviation.

### Visualizing MAC mediated killing by viability dye uptake

*S*. *aureus* Newman Δ*spa* was grown in phenol-free RPMI medium (Gibco) until it reached an OD_600_ ~1.0, washed once with PBS and resuspended in 3% BSA in PBS. They were blocked for 30 min at room temperature and then washed two times with PBS and resuspended in 0.05% BSA in phenol-free RPMI to an OD_600_ ~ 1. 200 μl of bacteria was mixed with 200 μl of NHS (Quidel), 200 μl C1q-depleted human serum (Quidel) or C5-depleted serum (Quidel) and samples were incubated for 0, 10 or 24 h at 37 °C with shaking. After incubation, propidium iodide (PI) was added to each sample to a final concentration of 1 μg/ml and incubated at room temperature for 5 min. Fluorescence was measured using a SpectraMax i3x plate reader (excitation 533 nm, emission 617 nm; Molecular Devices). Separate samples were prepared in triplicate for 0, 10 and 24 h analysis and results are plotted as mean with standard deviation.

### Production of bispecific antibodies (bsAbs)

BsAbs were produced in CHO cells after transfection with three expression plasmids encoding a target antigen binding IgG1 heavy chain, a target antigen binding light chain and either a C1q binding heavy chain or a C1q scFv-Fc that contains the H_435_R, Y_436_F mutations as described previously^17,47^. BsAbs were purified by differential protein A affinity chromatography.

### Surface plasmon resonance (SPR) binding analysis

Binding kinetics and affinities of C1q-recruiting bsAbs were assessed using surface plasmon resonance technology on a Biacore T200 or Biacore 8K instrument (GE Healthcare). A series of analyte concentrations were prepared in HBS-EP running buffer (0.01 M HEPES, 0.15 M NaCl, 3 mM EDTA and 0.05% surfactant Polysorbate 20) and injected at a flow rate of 30–50 μl/min for 2–2.5 min over flow cells (FCs) of Series S CM5 sensor chips immobilized with ligand molecules at various densities depending on assay formats. A capture sensor surface was prepared by covalently immobilizing with anti-human IgG F(ab’)2 fragment specific antibody (Jackson Immuno Research), NeutrAvidin (Thermo Fisher) or an anti-his monoclonal antibody (His capture kit, GE Healthcare,) to the chip surface using (1¬Ethyl-3-[3-dimethylaminopropyl]carbodiimide hydrochloride)/N-hydroxysuccinimide (EDC/NHS) coupling chemistry. Following surface activation, anti-human IgG F(ab’)2, NeutrAvidin or anti-his antibody in coupling buffer (0.1 M acetate buffer, pH 4.5) was injected over the activated chip surface until a resonance unit (RU) signal of about ~10000 RU (anti-human IgG F(ab’)2 polyclonal antibody), ~2000 RU (NeutrAvidin) or ~10000 RU (anti-his monoclonal antibody) was reached. The activated coupled chip surfaces were then washed and treated with 10 mM glycine-HCl, pH 1.5, to remove uncoupled residual proteins. In each format, all experiments were carried out at 37 °C.

In the first format (Supplementary Fig. 2a), antibodies were diluted into the running buffer and captured on the coupled anti-human IgG F(ab’)2 polyclonal antibody chip surface at ∼250 resonance units (RU) density, while the antigens were the analytes. Following the capture step, a range of concentration of test antigen (20.0 nM to 0.625 nM for C1q protein, and 90 nM to 1.11 nM for IsdB.6xHis protein) were individually injected over C1q-recruiting bsAb captured surfaces for 2–2.5 min. For all ligands, the association rate constant (ka) was determined from data obtained at multiple test ligand concentrations. The dissociation rate constant (kd), which is independent of test ligand concentration, was determined from the change in antigen-bound test ligand RU over time (~5–10 minutes) for C1q and IsdB.6xHis protein, respectively.

In the second format (Supplementary Fig. 2b), the biotin-C1q protein was captured on the coupled NeutrAvidin where the final ligand density was ~10 RU. Following the capture step, a range of concentrations of bsAb (500 nM to 7.8 nM), were individually injected over biotinylated C1q capture surfaces for 2 min. For all ligands, the ka was determined from data obtained at multiple test ligand concentrations. The kd was determined from the change in antigen-bound test ligand RU over time (~5 minutes) for C1q-recruiting bsAb respectively.

In the third format (Supplementary Fig. 2c), the IsdB.6xHis protein was captured on the coupled anti-his antibody chip where the final ligand density was ~350 RU. Following the capture step, a range of concentrations of bsAb (50 nM to 12.5 nM), were individually injected over IsdB.6xHis capture surfaces for 2.5 min. For all ligands, the ka was determined from data obtained at multiple test ligand concentrations. The kd was determined from the change in antigen-bound test ligand RU over time (~5 minutes) for C1q-recruiting bsAb, respectively. In addition, binding kinetics was performed for C1q protein (20 nM to 0.625 nM) flowing over complex IsdB.6xHis plus C1q-recruiting bsAb captured surface.

Specific Biacore kinetic sensorgrams were obtained by a double referencing procedure as described by Myszka *et al*.^48^. Because of the hexameric nature of C1q protein and the presence of multiple potential binding sites in the first format, definitive monovalent binding affinities for C1q protein and bsAb can be challenging to obtain. Very low immobilization densities were used to encourage monovalent binding, and the presence of such interactions were evaluated using a 1:1 Langmuir binding model. The data were then processed, and kinetic analyses performed using Scrubber software (version 2.0, BioLogic Software). The kd and ka were obtained via kinetic fitting, and the equilibrium dissociation constant (K_D_) was derived by taking the ratio of kd over ka calculated using the simplest 1:1 binding model.

### Antibody dependent C1q complement deposition on *S*. *aureus*

Antibodies were tested for complement deposition on *S*. *aureus* Newman in an ELISA based assay format. Briefly, *S*. *aureus* was grown in RPMI overnight, washed in PBS and adjusted to an OD_600_ of 0.25. Nunc MaxiSorp 96 well microtiter plates (ThermoFisher) were coated overnight with 100 μl of *S*. *aureus* culture per well. Plates were fixed with 2% PFA and blocked with 3% BSA in PBS prior to addition of a 1:3 serial dilution of antibody ranging from 0.14 nM – 33.3 nM for 1 h at 25 °C. After washing, 5% protein A/G adsorbed NHS (BioIVT) was added for 1.5 h at 37 °C. Goat anti-C1q antibody (final concentration 1 μg/ml; Genway Biotech, Inc.) was added to detect C1q deposition, followed by donkey anti-goat HRP secondary antibody (1:4000; Jackson ImmunoResearch) and chemiluminescent substrate (ThermoFisher). Luminescence was detected using a SpectraMax i3x plate reader (Molecular Devices). Luminescence values were analyzed by a four-parameter logistic equation over a 7-point response curve (GraphPad Prism) to calculate the binding EC_50_ of the antibodies. Results are plotted as mean ± standard deviation.

### Determination of C3b deposition on the surface of *S*. *aureus*

To remove IgG from serum, *S*. *aureus* was added to a final OD_600_ of 1.0 to NHS and incubated on ice for 30 min. The sample was centrifuged, and the pellet was discarded. Fresh *S*. *aureus* was added, and the serum was again incubated on ice for 30 min. After centrifugation, the supernatant was used as “preadsorbed serum.” *S*. *aureus* Newman Δ*spa* was grown in phenol-free RPMI medium (Gibco) to an OD_600_ ~1.0, washed 1x with PBS and resuspended in 3% BSA in PBS. Bacteria blocked for 30 min at room temperature (RT) and washed 2x with PBS were resuspended in 0.05% BSA in phenol-free RPMI to an OD_600_ ~ 1. 200 μl of bacteria was mixed with 200 μl of NHS (Quidel), preadsorbed serum, C1q-depleted human serum (Quidel), or media plus antibody at 100 μg/mL final concentrations and incubated for 1 h at 37 °C with shaking. After incubation, samples were washed 2x with 1% BSA in PBS and resuspended in 1% BSA in PBS containing 3 μg/ml anti-C3b antibody (clone 3E7; Biolegend). After incubating for 1 h at RT and washing 2x with 1% BSA in PBS, samples were resuspended in 1% BSA in PBS containing 2 μg/ml goat anti-mouse IgG antibody, Alexa Fluor 488 conjugated (Invitrogen) and incubated at RT for 45 min. 0.5 μM ToPro3 (ThermoFisher) was added for 15 min. Samples washed 2x with 1% BSA in PBS were resuspended in 1% BSA in PBS and analyzed on a BD FACSCanto II.

### *S*. *aureus* whole blood survival assays

Antibodies were assessed for bactericidal activity against *S*. *aureus* Newman in a whole blood survival assay^49^. Briefly, *S*. *aureus* Newman was grown in phenol-free RPMI overnight, washed in PBS, resuspended to a concentration of 1 × 10^8^ CFU/ml in PBS and then further serially diluted to a final concentration of 1 × 10^5^ CFU/ml. Counts for the bacterial input were determined by serial dilution and plating. In triplicate, 10 μl of the *S*. *aureus* suspension was mixed with 10–500 μg/ml test and control antibodies along with 100 μl of whole human blood (in sodium citrate as anti-coagulant) in microcentrifuge tubes. Where indicated, the blood was preincubated with C5-blocking Fab, isotype control Fab and cytochalasin D for 10 min at room temperature. The samples were incubated at 37 °C with shaking (100 rpm) for 24 h. After incubation, 100 μl of agglutination lysis buffer (PBS supplemented with 200U Streptokinase, 2 μg/ml RNase, 10 μg/ml DNase, 0.5% saponin, 100 μg trypsin per ml of PBS) was added to the samples and they were vigorously vortexed until the clot dissolved. 50 μl from each sample was serially diluted in PBS and plated onto LB agar plates for enumeration of CFUs. Results are plotted as mean with standard deviation.

### Antibody mediated complement activation in NHS

NHS (BioIVT and Quidel) or NHS containing 100 μg/ml control and test antibodies were incubated at 37 °C with shaking at 600 rpm for 1 h. C1q, C4, C4b, C2, C3, C3b, C5 and C5a levels were measured using the Milliplex human complement panels 1 and 2 from Millipore. Samples were diluted 1:200 for Panel 1 and 1:40,000 for Panel 2. Instructions provided in the manual were followed and samples were analyzed using a Luminex Flexmap 3D instrument.

### Copy number determination of *S*. *aureus* surface antigens

Standard curves for each antibody were generated using the Quantum Simply Cellular anti-human IgG beads (Bangs Laboratories, Inc.) according to the manufacturer’s protocol. Briefly, one drop of the reference microspheres was added to 50 μl FACS buffer [2% FBS (Gibco) in PBS]. Fluorochrome-conjugated antibodies specific to each surface antigen were added to the four standards at a saturating concentration (~450 nM). The microspheres were analyzed on a BD FACSCanto II flow cytometer and values were used to generate a standard curve converting MFI to antibody-binding capacity (ABC) using the Bangs Laboratories, Inc. quantitative analysis template, QuickCal. Next, the fluorochrome-conjugated antibodies were serially diluted in buffer starting at a concentration of 450 nM. To determine copy number of each surface antigen, *S*. *aureus* Newman Δ*spa* was grown in phenol-free RPMI medium overnight. The bacteria were washed twice with PBS and resuspended in FACS buffer. PI was added to each sample to a final concentration of 2.5 μg/ml and allowed to incubate with the bacteria for 2 min at room temperature. Bacterial viability was then determined by flow cytometry. 50 μl of bacteria were mixed with 50 μl of the serially diluted antibodies previously prepared. Samples were incubated on ice for 30 min and then 1 ml of ice-cold buffer was added. The samples were washed twice and resuspended in 200 μl of buffer. They were analyzed on a BD FACSCanto II using the same settings as utilized previously. The MFI values were converted to ABC using the microsphere standard curves for each antibody generated above. The copy number was taken at the saturating ABC value.

### Humanization of mouse C1q heads

#### Construction of bacterial artificial chromosomes (BACs) by bacterial homologous recombination and ligation

Using the VelociGene technology, bacterial homologous recombination was performed as described previously^50^. The mouse *C1qA*, *C1qB*, and *C1qC* genes were deleted and a *LacZ* gene was inserted under control of the mouse *C1qA* promotor. Chimeric C1q was produced by construction of unique targeting vectors from synthesized human sequences and mouse BAC DNA. The chimeric C1qA, C1qB and C1qC polypeptides contain human C1qA, C1qB and C1qC globular head domains that are essentially identical with the globular head domains of the corresponding human C1q polypeptides. The chimeras also contain N-terminal mouse stalk-stem regions that are essentially identical with the N-terminal stalk-stem regions of the endogenous mouse C1q polypeptide. The BAC sequences were confirmed by sequencing. DNA from the mouse BAC was modified to replace genomic DNA encoding portions of the mouse C1q genes, which are located in close proximity to one another on the reverse strand of mouse chromosome 4, with corresponding portions of human *C1qA*, *C1qB* and *C1qC*, respectively (human chromosome 1). A detailed description of the steps for constructing all BAC vectors is provided in Supplementary Fig. 7a–c (Human sequences are in blue, mouse sequences are in red, orange rectangles indicate selection cassettes, purple rectangles indicate Spectinomycin selection cassettes, cyan rectangles indicate Neomycin selection cassettes, yellow triangles indicate Frt sites, and purple triangles indicate LoxP sites). A list of primers for BAC production can be found in Supplementary Table 4.

#### Modification of ES cells and generation of mice

Targeting of ES cells (F1H4) was performed using the VelociGene method as previously described^50^. Derivation of mice from modified embryonic stem (ES) cells by eight-cell morula injection was as previously described^51^. The sequences of qPCR probes and primers used to screen for targeted ES cells and mice are included in Supplementary Table 5. All mice were housed and bred in specific pathogen-free conditions at Regeneron Pharmaceuticals. All mouse studies were overseen and approved by Regeneron’s Institutional Animal Care and Use Committee (IACUC) and performed in accordance with these IACUC guidelines.

### Quantification of C1q concentration in mouse serum using LC-MS/MS

Proteins from humanized mouse serum samples were denatured, reduced and subsequently alkylated and digested with trypsin. The digested peptide mixture was then separated by reversed-phase liquid chromatography using an Agilent 1290 Infinity II LC Systems with an ACQUITY UPLC BEH130 C18 column (2.1 × 50 mm, 1.7 µm; Waters). 0.1% formic acid in water and 0.1% formic acid in acetonitrile were used as mobile phase A and mobile phase B, respectively. Signature peptides unique to human C1q protein sequences were selected from each of the individual C1q subunits (A, B and C) as surrogate peptides for C1q quantification. Calibration curves for the surrogate peptide from each C1q subunit were generated using a series of concentrations of C1q protein standard (Quidel), which allows for the quantitation of total C1q by any of the three subunits based on the assay response of the respective surrogate peptides. Data acquisition by LC-MS/MS under selected reaction monitoring (SRM) mode was performed using an Agilent 6495 TripleQuad mass spectrometer with Agilent Jet Stream electrospray ionization source. Pre-selected mass to charge ratio (m/z) of precursor and product ion pair of each surrogate peptide were fragmented with optimized collision energy and detected in the mass spectrometer. Agilent LC/MS Data Acquisition for 6400 Series Triple Quadrupole, version B.08.00, was used to run the LC/MS system. Agilent MassHunter Quantitative Analysis, version B.06.00, was used for data analysis. In this assay, all standards, controls and test samples were prepared in parallel. Results are plotted as median with interquartile range.

### CH50 hemolysis assay

Sheep red blood cells (SRBCs; Complement Technologies, Inc.) were washed in GVB++ buffer (Complement Technologies, Inc.) and resuspended at 1 × 10^9^ cells/ml. The SRBCs were then opsonized with rabbit anti-sheep hemolysin. Sensitized SRBCs were diluted to 2 × 10^8^ cells/ml in GVB++ buffer prior to using in hemolysis assay. Serum from wild-type (n = 5) and C1q humanized (n = 4) mice was collected at seven to nine weeks of age. Mouse serum was serially diluted in a 6 point, 2-fold dilution series from 1/5 to 1/160 with GVB++ buffer (100 μl diluted serum/well). Immediately, 100 μl of sensitized SRBCs (at 2 × 10^8^ cells/ml) were added, for a total volume of 200 μl, and incubated 1 h at 37 °C. After incubation, cells were pelleted by centrifugation at 1250 × g at 4 °C. A total of 100 μl of the supernatant was transferred to a fresh 96-well flat bottom plate and read at 412 nm using a SpectraMax M5 microplate reader and SoftMax Pro software (Molecular Devices). The hemolytic activity was calculated as follows:

$$\frac{{{\rm{OD}}}_{412}\,{\rm{of}}\,{\rm{experimental}}\,{\rm{samples}}}{{{\rm{OD}}}_{412}\,{\rm{at}}\,{\rm{Maximum}}\,{\rm{cell}}\,{\rm{lysis}}\,({\rm{cells}}\,{\rm{treated}}\,{\rm{with}}\,100\,{\rm{\mu }}{\rm{l}}\,{\rm{water}})}\times 100$$

Data represented are single points (duplicates not run).

### Disseminated *S*. *aureus* infection model

Humanized C1q mice were stratified by weight on day 0 and then infected intraperitoneally^52^ with 200 μl volume of *S*. *aureus* Newman (1.5 × 10^8^ CFU/mouse), *S*. *aureus* CA-127 (1.5 × 10^8^ CFU/mouse), or *S*. *aureus* MW2 (1.3 × 10^8^ CFU/mouse). Overnight *S*. *aureus* cultures were grown in tryptic soy broth (TSB), diluted 1:100 in fresh TSB and grown to log phase (OD_600_ ~ 1) at 37 °C with shaking. The culture was washed 3 times with PBS and adjusted to the desired density for infection. Where indicated, antibiotics were administered starting 18 h following infection. Mice were treated with 100 μl volume of 110 mg/kg of vancomycin twice daily administered subcutaneously, 50 mg/kg daptomycin once daily administered subcutaneously or 80 mg/kg linezolid twice daily administered orally on Days 1, 2 and 3. 24 h post infection, mice were administered with a single dose of the indicated antibody or PBS in a 100 μl volume, intraperitoneally. Mice were weighed daily until day 4 post infection and change in weight was recorded. Results are plotted as the mean percent weight change of each group of mice. Mice were euthanized on day 4 and kidneys, heart, liver, lungs and spleen were collected to determine organ burden. Briefly, the organs were homogenized in 5 ml PBS using program “Multi-C” on a gentleMACS Octo Dissociator (Miltenyi Biotec). Homogenate was then diluted in PBS and 10-fold serial dilutions were plated on LB agar plates which were incubated at 37 °C overnight. Resultant colonies were counted, and results were reported as CFU/gram tissue. The limit of detection for the assay is 5 × 10^2^ CFU/g. Organ burden for each mouse is shown along with the median value for the group.

### Determination of antibody binding to target cells using ELISA

#### *P. aeruginosa*

An overnight *P*. *aeruginosa* PAO1 culture was grown in LB, diluted 1:50 in fresh LB and grown to OD_600_ = ~1 at 37 °C with shaking (240 rpm). The culture was washed once with PBS and diluted to OD_600_ = 0.25. Nunc MicroSorp 96-well plates (ThermoFisher) were coated with 100 μl per well of the *P*. *aeruginosa* suspension and incubated overnight at 4 °C. The following morning, plates were washed once with wash buffer (0.002 M Imidazole buffered saline, 0.02% Tween-20) and blocked for 2 h at 25 °C with 200 μl of blocking buffer (3% BSA in PBS). Plates were washed once, and titrations of the bispecific, bivalent and isotype-matched control antibodies ranging from 333 nM – 5.64 pM with 1:3 serial dilutions in assay dilution buffer (ADB: 0.5% BSA, 0.05% Tween-20 in PBS) were added to the bacteria-containing wells and incubated for 1 h at 25 °C. Wells were washed three times and then incubated with anti-human IgG HRP conjugated detection antibody (Jackson ImmunoResearch; 1:4000) in ADB for 1 h at 25 °C followed by chemiluminescent substrate (ThermoFisher). Luminescence was detected on a SpectraMax i3x plate reader (Molecular Devices). Luminescence values were analyzed by a three-parameter logistic equation over a 11-point response curve (GraphPad Prism).

#### Raji cells

To determine the binding of the antibodies to CD20 expressed on Raji cells, cells at a density of 1 × 10^7^/ml fixed in 2% PFA were used. The fixed cells were blocked with 3% BSA for 1 h at room temperature, washed with wash buffer (0.002 M imidazole buffered saline, 0.02% Tween-20), and 100 μl of the washed cells were aliquoted into Nunc untreated plates. The CD20 bivalent, anti-CD20 x anti-C1q and anti-C1q bivalent antibodies were serially diluted 1:2 in ADB (0.5% BSA, 0.05% Tween-20 in PBS), starting from 250 nM to 0.015 nM and added to the Raji cells in a 100 μl volume and incubated for 1 h at room temperature. After washing the cells three times, 100 μl of anti-human IgG HRP conjugated detection antibody (Jackson ImmunoResearch; 1:4000) in ADB was added to the cells and incubated at room temperature for 1 h. The cells were washed three times, transferred to a Nunc MaxiSorp™ black plate, and incubated for a further 10–15 min with the chemiluminescent substrate before reading the plate on a SpectraMax i3x plate reader (Molecular Devices). The results, plotted as RLUs of binding on the y axis vs. log(M) antibody concentration on the x axis, were analyzed using nonlinear regression (4-parameter logistics) with GraphPad Prism software to determine an EC_50_ value of antibody binding.

### Determination of antibody binding to Jurkat cells using flow cytometry

Flow cytometric analysis was used to investigate binding of anti-GITR x anti-C1q bsAb and the anti-GITR bivalent antibody to target cells expressing human GITR. Binding was investigated using an engineered cell line, Jurkat/GITR/CD20. Briefly, cells (300,000 cells/well) were incubated for 30 min at 4 °C with LIVE/DEAD Fixable Green Dead Cell Stain (ThermoFisher) according to manufacturer’s instructions to discriminate between live and dead cells. The cells were then washed twice with cold PBS containing 2% FBS and incubated for 30 min at 4 °C with serial dilutions (1 pM to 1 μM) of anti-GITR x anti-C1q, anti-GITR bivalent or IgG1 isotype control antibody. After incubation, the cells were washed twice with cold PBS containing 2% FBS, and then incubated for 1 h at 4 °C with Alexa-Flour 647-conjugated goat anti-human IgG antibody (Jackson ImmunoResearch) at a final concentration of 2 μg/ml. fixed in BD Cytofix Buffer according to manufacturer’s instructions, washed, re-suspended in PBS and analyzed by flow cytometry on an iQue Screener flow cytometer (Intellicyt). Unstained and secondary antibody alone controls were also tested for both cell lines. The results were analyzed using FlowJo (TreeStar, Inc.) software and geometric mean fluorescence for viable cells were determined. The results were analyzed using nonlinear regression (4-parameter logistics) with GraphPad Prism software to obtain EC_50_ values for binding to cells.

### *P*. *aeruginosa* whole blood survival assay

Antibodies were assessed for bactericidal activity against *P*. *aeruginosa* PAO1 in a whole blood survival assay. Briefly, *P*. *aeruginosa* PAO1 was grown in TSB overnight, washed in PBS and resuspended to a concentration of 1 × 10^8^ CFU/ml in PBS. They were further serially diluted to a final concentration of 1 × 10^5^ CFU/ml. Actual counts for the bacterial input were determined by serial dilution and plating. 10 μl of the *P*. *aeruginosa* suspension was mixed with 10, 100, or 500 μg/ml bsAb or an equimolar amount of bivalent antibody (12, 120 or 600 μg/ml respectively), along with 100 μl of whole human blood (in sodium citrate as anti-coagulant), in triplicates, in microcentrifuge tubes. The samples were incubated at 37 °C with shaking (100 rpm) for 24 h. After incubation, 100 μl of agglutination lysis buffer (PBS supplemented with 200U Streptokinase, 2 μg/ml RNase, 10 μg/ml DNase, 0.5% saponin, 100 μg/ml trypsin) was added to the samples and they were vigorously vortexed until the clot dissolved. 50 μl from each sample was serially diluted in PBS and plated onto LB agar plates for enumeration of CFUs. Results are plotted as mean with standard deviation.

### Complement dependent cytotoxicity (CDC) assays

CDC assays were used to test the efficacy of antibodies in eliciting complement mediated killing of target cells.

#### Raji cells

Raji cells were seeded onto a 96-well white assay plates with clear bottom at 10,000 cells/well in 1% BSA in phenol free RPMI medium. The test antibodies (anti-CD20 x anti-C1q bsAb, anti-CD20 bivalent hIgG1 antibody, Rituximab and anti-C1q bivalent hIgG1 antibody) were serially diluted 1:8 from 100 nM to 0.004 nM and added to the cells, along with human serum at a final concentration of 5%. Cells were incubated for 1 h at 37 °C and in 5% CO_2_ and cytotoxicity was measured using the CytoTox-Glo reagent (Promega) according to the manufacturer’s instructions. Results are plotted as mean ± standard deviation.

#### Jurkat cells

The ability of the anti-GITR x anti-C1q antibody to mediate CDC in the presence of NHS was evaluated in engineered Jurkat/hGITR/hCD20 target cells. NHS was added at a final serum concentration of 5% to target cells seeded at 5,000 cells/well in triplicate rows of a 96-well white plate. Serial dilutions of anti-GITR x anti-C1q antibodies ranging from 0.5 pM to 500 nM were then added to target cells. Cells were incubated for 3.5 h at 37 °C and 5% CO_2_ and cytotoxicity was measured using the CytoTox-Glo reagent (Promega) according to the manufacturer’s instructions. Results are plotted as mean ± standard deviation.

#### U937 cells

The ability of the bispecific antibody to mediate CDC against U937, an Fc-receptor bearing monocyte-like cell line, in the presence of NHS was evaluated. NHS was added at a final serum concentration of 5% to target cells seeded at 10,000 cells/well in triplicate rows of a 96-well white plate. Serial dilutions of anti-IsdB x anti-C1q antibodies ranging from 1 μM to 100 nM were then added to target cells. Cells were incubated for 3.5 h at 37 °C and 5% CO_2_ and cytotoxicity was measured using the CytoTox-Glo reagent (Promega) according to the manufacturer’s instructions. Results are plotted as mean ± standard deviation.

#### CytoTox-Glo assay

Briefly after incubation with serum, samples were equilibrated to room temperature for 30 min. CytoTox-Glo reagent was prepared and added to each well. To determine the maximal target cell lysis signal, untreated target cells alone were lysed with digitonin prior to addition of the chemiluminescent substrate. Luminescence was measured from each well. The percent cytotoxicity was calculated as follows:

$$\frac{{\rm{Experimental}}\,{\rm{Signal}}-{\rm{Spontaneous}}\,{\rm{Background}}\,{{\rm{Signal}}}_{({\rm{target}}{\rm{cells}}+{\rm{serum}})}}{{\rm{Max}}\,{{\rm{Signal}}}_{({\rm{target}}{\rm{cells}}+{\rm{digitonin}})}-{\rm{Spontaneous}}\,{\rm{Background}}\,{{\rm{Signal}}}_{({\rm{target}}{\rm{cells}})}}\times 100$$

### Flow cytometry-based CDC assays

#### Raji cells

Raji cells were washed with PBS and resuspended in phenol free RPMI to a final concentration of 4 × 10^6^ cells/ml. Test antibodies (anti-CD20 x anti-C1q bsAb, anti-CD20 bivalent hIgG1 antibody, Rituximab and anti-C1q bivalent hIgG1 antibody) were added to cells at a final concentration of 66, 33, 6.6, 3.3, 0.66 and 0.33 nM. 50% NHS was added. Cells were incubated for 30 min at 37 °C and in 5% CO_2_. Samples were washed twice with 2% BSA in PBS. After resuspending in 2% BSA in PBS, 1 μg/ml PI was added, and samples were analyzed on a BD FACSCanto II.

#### Jurkat cells

Jurkat/hGITR/hCD20 cells were washed with PBS and resuspended in phenol free RPMI to a final concentration of 4 × 10^6^ cells/ml. Test antibodies (anti-GITR x anti-C1q bsAb, anti-GITR bivalent antibody, and anti-FelD1 bivalent hIgG1 isotype control) were added to cells at a final concentration of 400, 200, 100, 50, and 25 nM. 50% NHS was added. Cells were incubated for 30 min at 37 °C and in 5% CO_2_. Samples were washed twice with 2% BSA in PBS. After resuspending in 2% BSA in PBS, 1 μg/ml PI was added, and samples were analyzed on a BD FACSCanto II.

#### U937 cells

Cells were washed with PBS and then resuspended in 1% BSA in phenol free RPMI to a final concentration of 4 × 10^6^ cells/ml. Antibody was added to a final concentration of 100 nM followed by 50%. The samples were incubated for either 30 min or 1 h at 37 °C and 5% CO_2_. Samples were washed twice with 2% BSA in PBS. After resuspending in 2% BSA in PBS, 1 μg/ml PI was added and samples were analyzed on a BD FACSCanto II.

### Determination of membrane complement inhibitor levels by flow cytometry

Flow cytometric analysis was used to characterize the CD55 and CD59 expression levels on Jurkat and Raji cells. Briefly, cells were incubated at 4 °C with BD APC anti-CD55, BD FITC anti-CD59, or isotype matched control antibodies. After incubation, the cells were washed with cold PBS containing 2% FBS, and then fixed in 4% formaldehyde in PBS, washed, re-suspended in PBS, and analyzed by flow cytometry on an Accuri C6 flow cytometer. Unstained controls were also tested for both cell lines. The results were analyzed using FlowJo (TreeStar Inc.) software and geometric mean fluorescence for viable cells were determined.

## Supplementary information

Supplementary Information
