# Supplementary material for: A novel bispecific antibody platform to direct complement activity for efficient lysis of target cells
Source: Sci Rep. 2019 Aug 19;9:12031. doi: 10.1038/s41598-019-48461-1 (PMC6700171; doi:10.1038/s41598-019-48461-1)
Supplement: Supplementary file 1 — Supplementary Information [file 41598_2019_48461_MOESM1_ESM.pdf]

A novel bispecific antibody platform to direct complement activity for efficient lysis of target cells

Short Title: C1q-recruiting bispecific antibodies lead to target cell cytotoxicity

Jonathan W. Cruz[a], Ermelinda Damko[a], Bhavika Modi[a], Naxin Tu[a], Karoline Meagher[a], Vera Voronina[a], Hans Gartner[a], George Ehrlich[a], Ashique Rafique[a], Robert Babb[a], Priya Aneja[a], Terra B. Potocky[a], Amanda D'Orvilliers[a], Alida Coppi[a], Sook Yen E[a], Haibo Qiu[a], Courtney M. Williams[a], Brandy L. Bennett[a], Gang Chen[a], Lynn Macdonald[a], William Olson[a], John C. Lin[a], Neil Stahl[a], Andrew J. Murphy[a], Christos A. Kyratsous[a], Brinda C. Prasad[a1]

[a] Regeneron Pharmaceuticals Inc., Tarrytown, NY 10591

[1]Corresponding Author: brinda.prasad@regeneron.com

Phone: 914-847-1023

Supplementary Figure Legends

Supplementary Figure 1 A complete terminal complement pathway is necessary for NHS mediated reduction in the growth of *S. aureus* as measured using BacTiter-Glo assay. The effect of human serum on growth of *S. aureus* was measured using serum killing assays. S. *aureus* was incubated with 50% of the indicated sera or medium for 24 h. After incubation, bacterial concentration was measured using the BacTiter-Glo assay, a luminescent assay which measures the number of viable cells in a culture via ATP release. Increased luminescence corresponds to higher concentrations of bacteria. Results are plotted as mean with standard deviation. *** $P < 0.001$, **** $P < 0.0001$, one-way ANOVA with Dunnett's test showing significance compared to the NHS sample.

Supplementary Figure 2 Kinetic analysis of anti-IsdB x anti-C1q bsAb binding to IsdB and C1q protein was performed under various formats using Biacore. Binding analysis was tested in various 2-fold dilution series, the association phase of ligand was monitored at 50 µl/min for 2-3 minutes over each of the captured surfaces. Representative diagrams of the formats are shown. (**a**) Anti-IsdB x anti-C1q bsAb (314 RU) was captured on an anti-human Fc coupled chip surface. Anti-IsdB x anti-C1q bsAb binding to monomeric his-tagged IsdB was (IsdB.his) measured at IsdB.his concentrations of 90, 45, 22.5, 11.2, 5.6 and 2.8 nM. (**b**) Biotinylated human C1q (24 RU) was captured on a neutravidin coupled chip surface. Biotinylated human C1q binding to anti-IsdB x anti-C1q bsAb was measured at bsAb concentrations of 200, 100, 50, 25, 5 and 1 nM, (**c**) IsdB.his (302 RU) was captured on an anti-his mAb coupled chip surface, followed by capturing anti-IsdB x anti-C1q bsAb (102 RU). Binding to human C1q protein at 20, 10, 5 and 2.5 nM are shown as black lines. The data were globally fit to a 1:1 binding with mass-transfer interaction model using Scrubber 2.0c. Kinetic fits from the analyses are overlaid on the binding data in red.

Supplementary Figure 3 C3b deposits on the surface of *S. aureus* in the presence of NHS. An anti-C3b antibody was used to detect complement deposition on the surface of *S. aureus* in the presence of NHS and the indicated antibodies. To remove anti-*S. aureus* antibodies, the serum for one set of samples was preadsorbed to *S. aureus*. Results were analyzed using flow cytometry and plotted as mean with standard deviation.

Supplementary Figure 4 The anti-IsdB x anti-C1q bsAb predominantly recruits complement through the C1q-binding arm and mediates cell killing only when directed to the target cell via the anti-IsdB targeting arm. (**a**) A whole blood assay was performed with two different isotypes of a control or anti-IsdB x anti-C1q antibody. The hIgG1 antibodies are able to recruit C1q through both the Fc and Fab portion of the molecule, whereas the hIgG4 antibodies are less capable of recruiting C1q through the Fc portion of the molecule. (**b**) A whole blood assay was performed with the *S. aureus* targeted anti-IsdB x anti-C1q bsAb and two bsAbs, anti-EGFRvIII x anti-C1q and anti-CD20 x anti-C1q, which do not recognize *S. aureus* surface antigens. Results are plotted as mean with standard deviation. * $P < 0.05$, *** $P < 0.001$, one-way ANOVA showing samples significantly different from the anti-IsdB x anti-C1q hIgG1 treated sample.

Supplementary Figure 5 In the absence of target, anti-C1q x anti-IsdB antibody does not activate complement. Luminex was used to measure the concentration of complement proteins (C1q, C4, C2, C3, and C5) as well as cleavage products (C4b, C3b, and C5a) present in serum after 1 h incubation with the anti-IsdB x anti-C1q bsAb, a control bivalent antibody, a control bsAb, or serum alone. NHS from two different vendors (Quidel and BioIVT) were used. Results are plotted as mean with standard deviation.

Supplementary Figure 6 Incubation of Fc receptor bearing cells with anti-IsdB x anti-C1q bsAb does not result in cytotoxicity. (a) A CDC assay was performed using CytoTox-Glo (Promega) reagent to determine if bsAb-C1q immune complexes could bind to Fc receptor-bearing U937 cells resulting in cytotoxicity. The cells were incubated with 1, 10 or 100 nM antibody in 5% NHS. Results are plotted as mean with standard deviation. (b) The assay was repeated with 50% NHS and uptake of the cell impermeant dye propidium iodide (PI) by dead cells was determined. Samples were analyzed by flow cytometry and percent dead cells (PI positive) is shown.

Supplementary Figure 7 Schematic representation of the production of C1q humanized mice. (**a**) Schematic representation (not to scale) of the method of deleting of the mouse *C1qA*, *C1qB* and *C1qC* genes (mouse genes are indicated with "m" before the gene label). Exons of the three C1q genes are labeled below the diagram (e.g., E1, E2, and E3). Bac, bacterial artificial chromosome; BHR, bacterial homologous recombination; EP, electroporation; HET,

heterozygous; CM, chloramphenicol; lox, loxP site; pgk-Neo, neomycin selection cassette. (**b**) Schematic representation (not to scale) of the creation of a humanized mouse C1q targeting vector, with chimeric human/mouse genes inserted by digestion/ligation and/or bacterial homologous recombination (BHR) into the mouse BAC genes (mouse genes are indicated with "m" before the gene label). Exons of the three C1q genes are labeled below the diagram (e.g., E1, E2, and E3). Selected restriction enzyme locations are indicated. CM, chloramphenicol; lox, loxP site; Ub-Hyg, hygromycin selection cassette; p, polyA tail; Spec, spectinomycin. (**c**) Schematic representation (not to scale) of the electroporation (EP) of a large targeting vector containing all three mouse/human chimeric C1q genes into mouse C1q KO HET ES cells. Exons of the three C1q genes are labeled below the diagram (e.g., E1, E2, and E3). Lox, loxP site; Ub-Hyg, hygromycin selection cassette; pgk-Neo, neomycin selection cassette; p, polyA sequence. Sequence junctions between mouse, human or cassette sequences are indicated with a line and a SEQ ID number for that respective sequence is shown below each junction. Human sequences are in blue, mouse sequences are in red.

Supplementary Figure 8 Anti-IsdB x anti-C1q bsAb reduces the bacterial burden of several strains of *S. aureus* in multiple mouse organs. (**a**) C1q humanized mice were infected with 1.4 x 10^8 CFU *S. aureus* CA-127 (a MRSA strain). One day after infection, mice were treated with the indicated antibody. Weights were recorded each day for four days and percent weight change compared to day 0 is shown (left). Mean weight change for each group of mice is plotted. On day 4, kidneys were harvested, dissociated, and serially diluted. Organ burden was determined by plating (right). (**b-e**) C1q humanized mice were infected with either 1.5 x 10^8 CFU/mouse *S. aureus* Newman (an MSSA strain) or 1.4 x 10^8 CFU/mouse *S. aureus* CA-127 (an MRSA strain) on day 0. One day after infection, mice were treated with the indicated antibody intraperitoneally. On day 4 heart (**b**), liver (**c**), lungs (**d**) and spleen (**e**) were harvested, dissociated and serially diluted to enumerate bacterial load. The organ burden of each mouse is indicated along with the median value for each group.

Supplementary Figure 9 Antibody binding to fixed and unfixed Raji cells. To determine if the process of fixing affected antibody binding to Raji cells we tested binding of anti-C1q bivalent antibody, anti-CD20 bivalent antibody, and anti-C1q x anti-CD20 bsAb to both fixed and unfixed Raji cells. Curves with colored symbols show binding to fixed cells. Curves

with black symbols show binding to unfixed cells. A table of EC_{50} values (in molarity) of binding is shown below.

Supplementary Figure 10 C1q-recruiting bsAb induce CDC in the presence of 50% NHS. To measure the cytotoxic effects of the bsAbs with physiological serum concentrations of 50%, Raji (**a**) and Jurkat/hGITR/hCD20 (**b**) cells were incubated with the indicated antibody and 50% NHS. Dead cells were labeled with the cell impermeant PI dye and analyzed using flow cytometry. A table of EC_{50} values (in molarity) of cytotoxicity is shown for each antibody tested.

Supplementary Table 1 C1q-recruiting bsAbs that recognized *S. aureus* surface antigens bind to C1q with similar affinity and kinetics. Each of the bsAbs was Fc-captured on the surface of a Biacore chip. 5 nM human C1q was then injected across the surface and binding properties were measured.

Supplementary Table 2 C1q-recruiting bsAbs bind their respective *S. aureus* surface antigens. Purified *S. aureus* surface antigens (IsdB and IsdA) were captured on the surface of a Biacore chip. 50 nM bsAb or 90 nM bivalent antibody was then injected across the surface of the chip. Binding parameters of the antibodies were measured. For antigens that could not be expressed and purified (PNAG, ClfA and Protein A) binding of the antibodies to *S. aureus* was determined by ELISA. Antibody dependent recruitment of C1q to the surface of *S. aureus* was determined in an ELISA format.

Supplementary Table 3 *S. aureus* target antigens are present at different densities on the surface of the bacterium. Copy number of various surface antigens present on *S. aureus* Newman Δ*spa* grown in RPMI was determined using flow cytometry. By using beads with known antibody-binding capacity (ABC), and saturating fluorochrome-conjugated antibody concentrations (150 nm), the number of each antigen was determined. Results are expressed as the ratio of specific antibody bound compared to an isotype control.

Supplementary Table 4 List of primers used in the cloning and production of BAC vectors

Supplementary Table 5 List of probes and primers used in qPCR screening of mouse embryonic stem cells

Supplementary Table 6 C1q-recruiting bsAbs bind to C1q with similar affinity and kinetics. Each of the bsAbs was Fc-captured on the surface of a Biacore chip. 5 nM human C1q was then injected across the surface and binding properties

were measured.

Supplementary Table 7 anti-GITR x anti-C1q binds the GITR antigen. Purified GITR was captured on the surface of a Biacore chip. 50 nM bsAb was then injected across the surface of the chip. Binding parameters of the antibodies were measured. For antigens that could not be expressed and purified (Psl and CD20) binding of the antibodies to respective whole cell was determined by ELISA (see **Table 2**).

Supplementary Table 8 Raji B-cells and Jurkat T-cells express different levels of the complement inhibitors CD55 and CD59. Relative levels of CD55 and CD59 were determined by binding fluorescently-labeled antibodies specific for each protein to both cell types and analyzing by flow cytometry. Results are expressed as the ratio of specific antibody binding compared to an isotype control.

Supplementary Figure 1

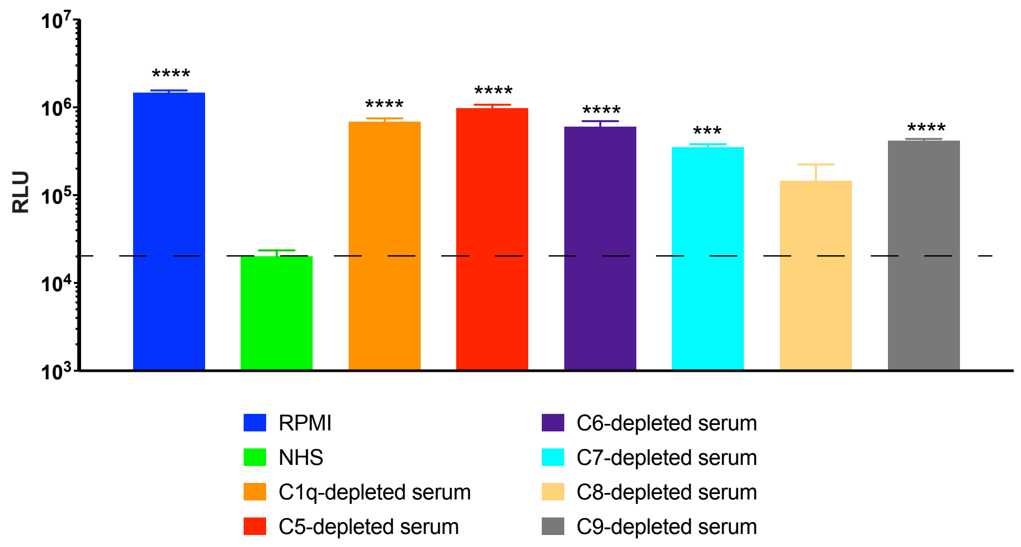

Supplementary Figure 2

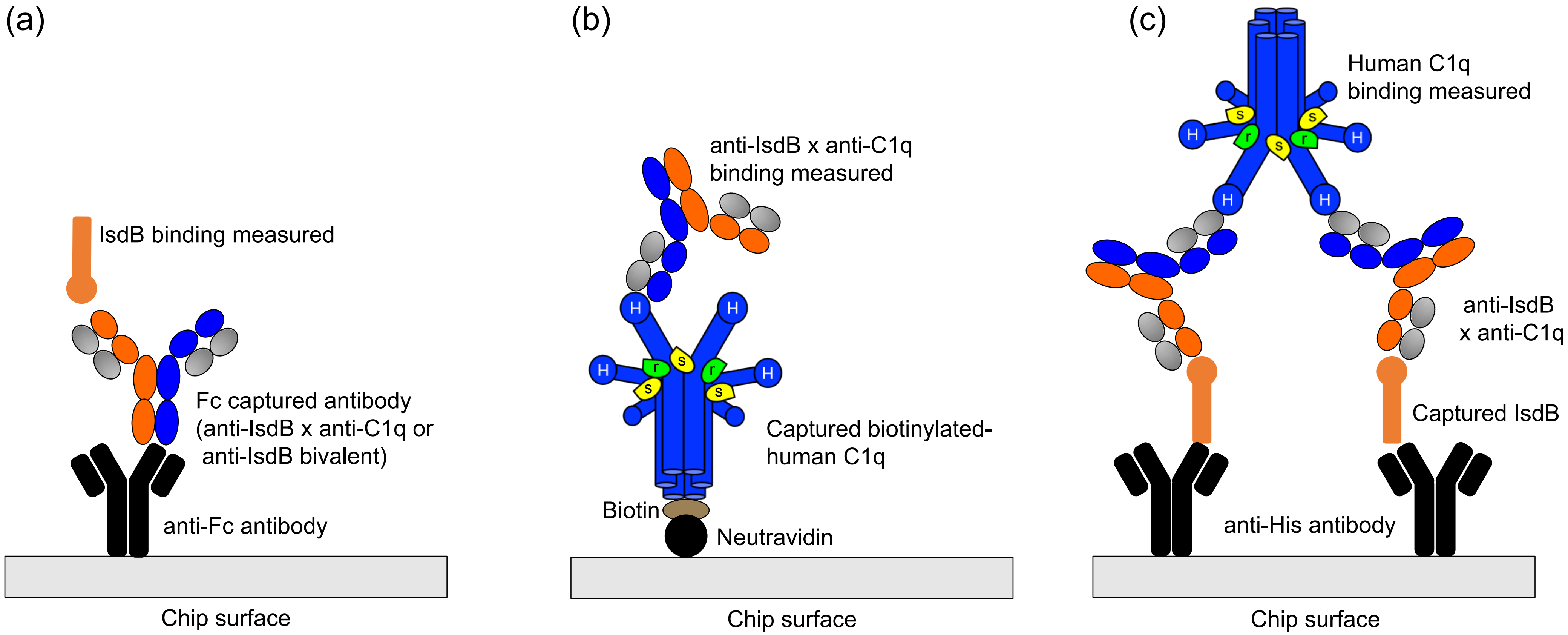

Supplementary Figure 3

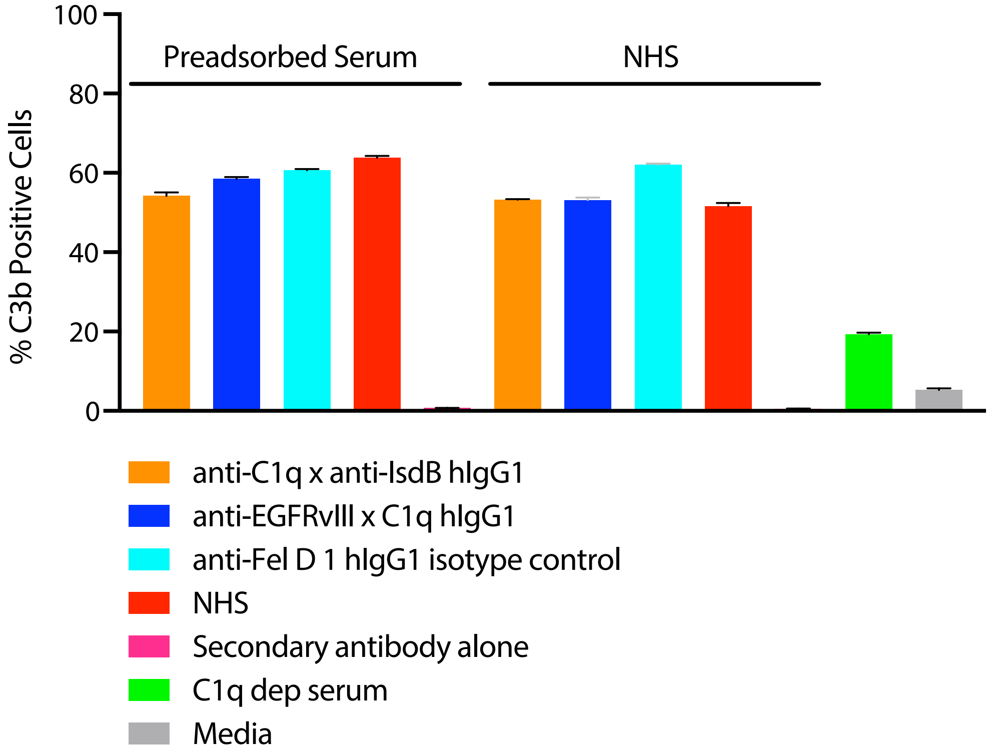

Supplementary Figure 4

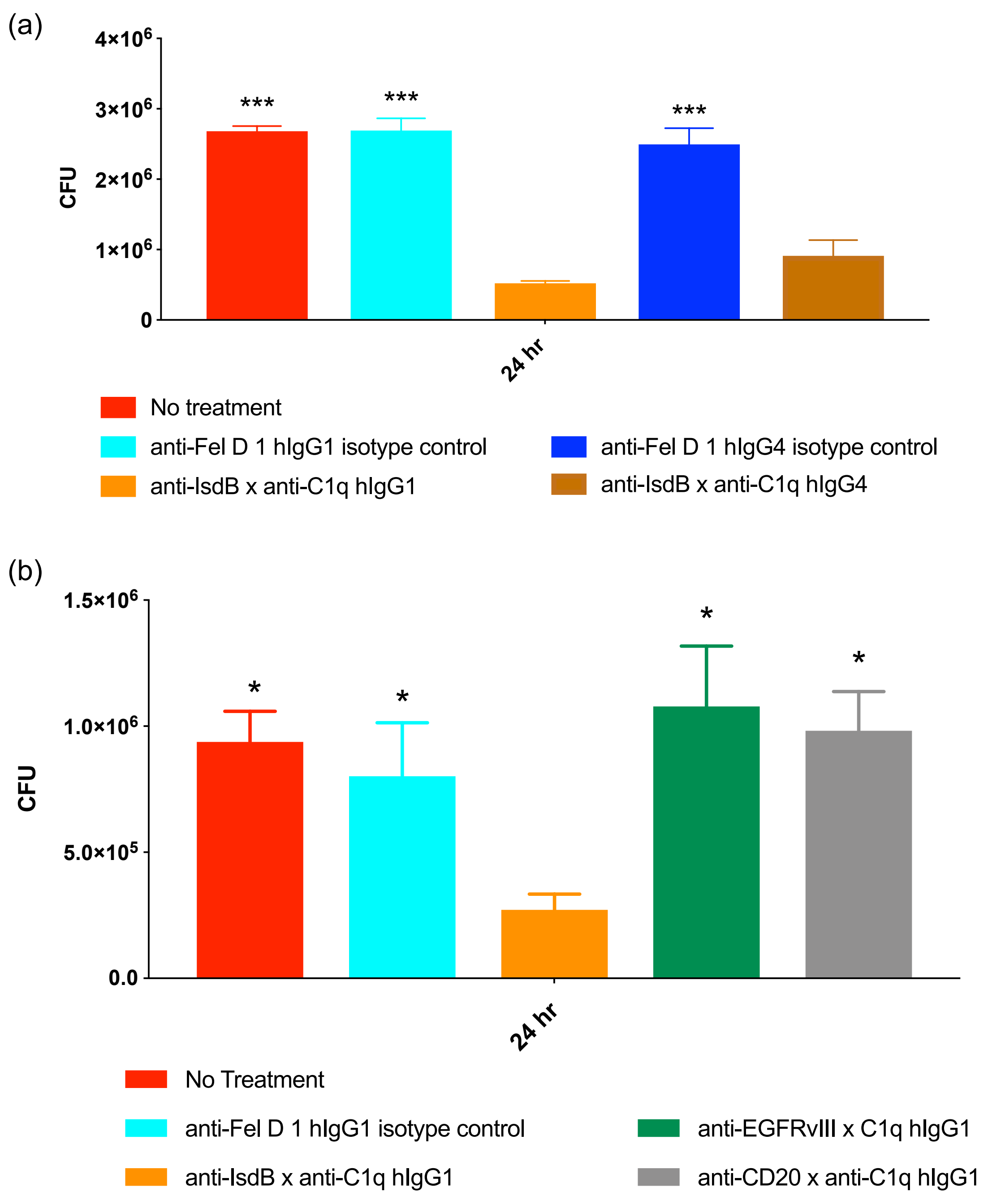

Supplementary Figure 5

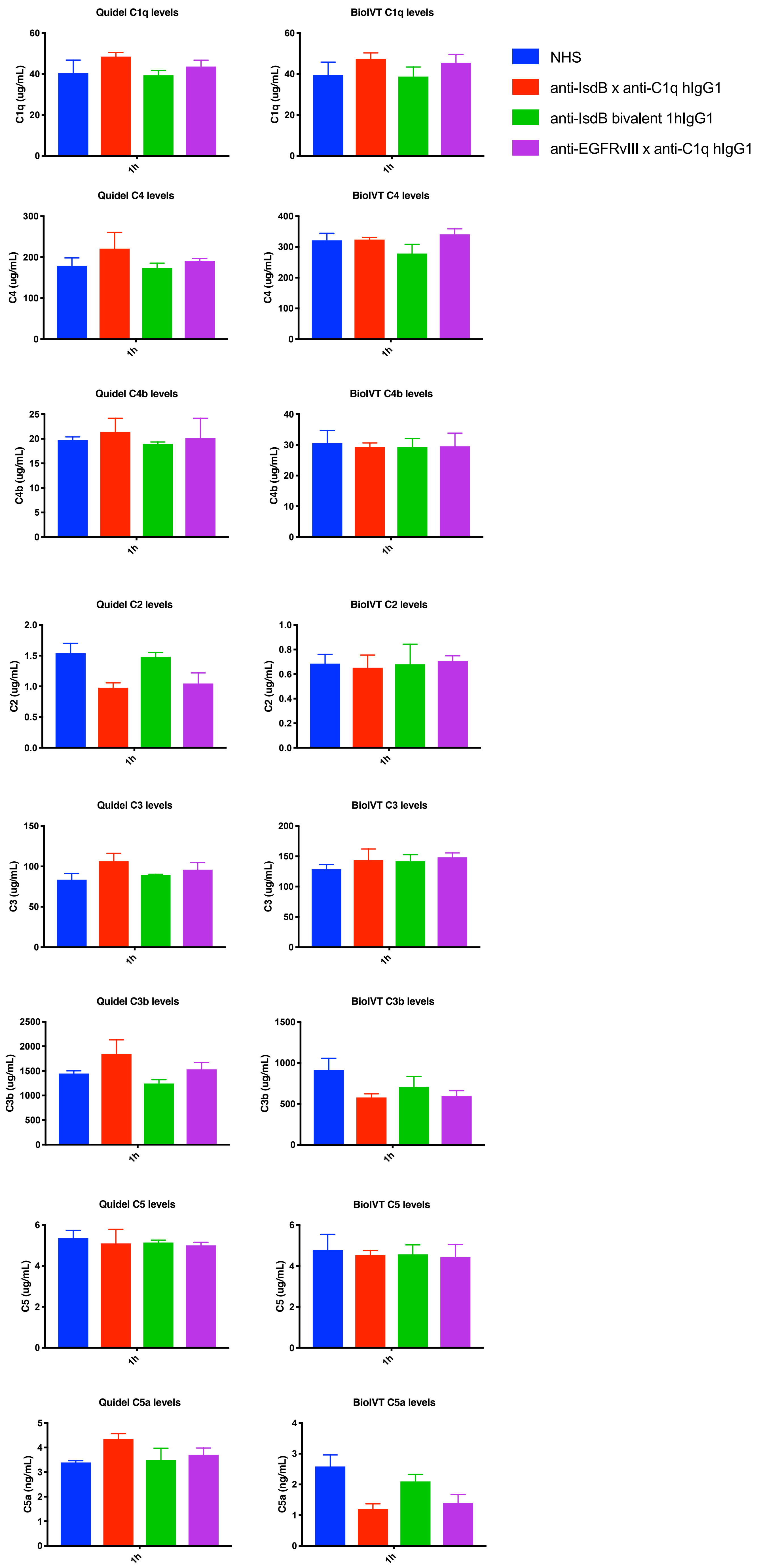

Supplementary Figure 6

(a)

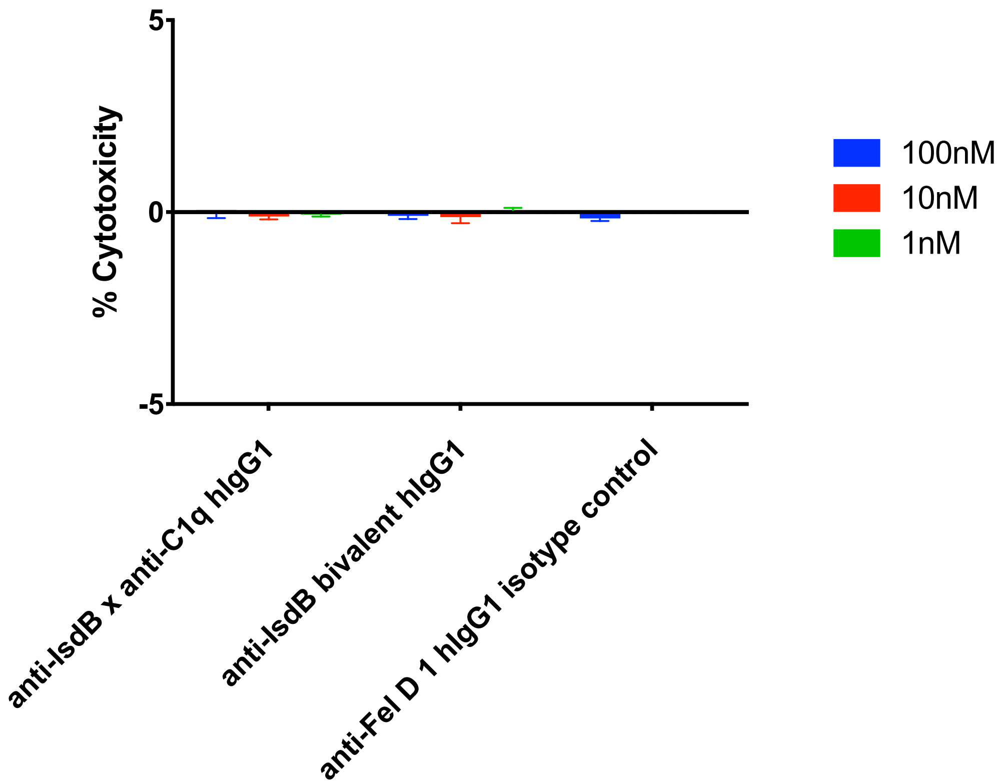

(b)

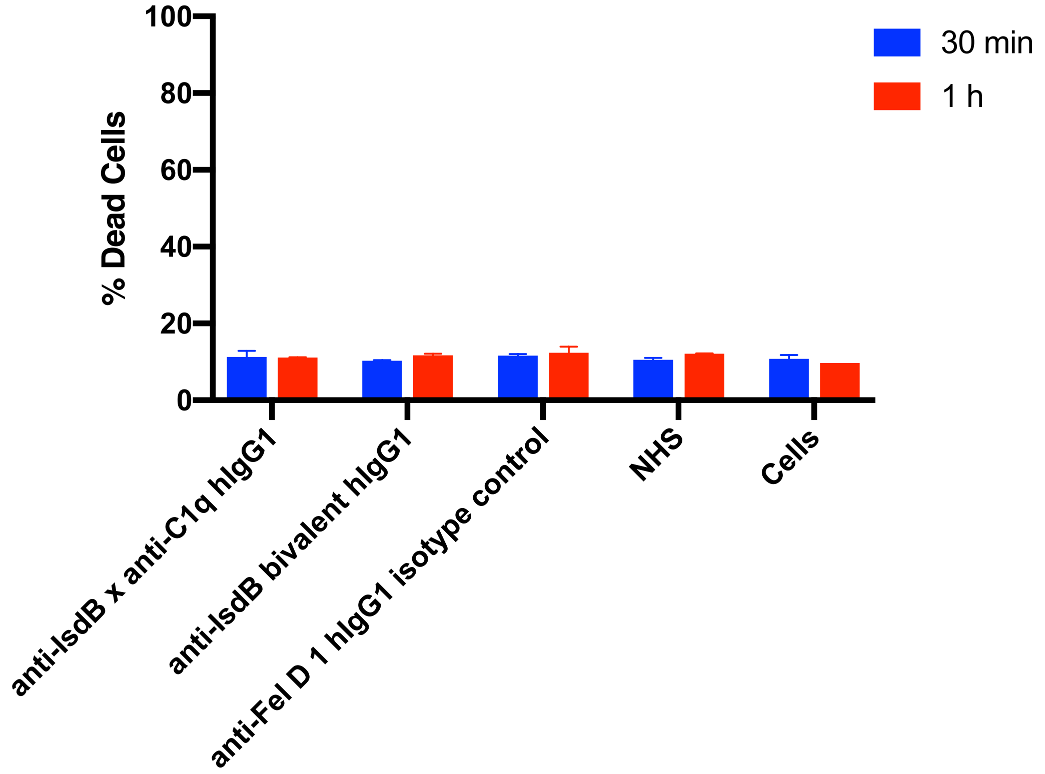

Supplementary Table 1 Binding parameters for 5 nM C1q binding as the test ligand to antibody captured chip surfaces

Targeting Arm	Capture Surface	Biacore kinetic parameters for binding at 37 °C				C1q recruitment to the surface of *S. aureus*	
		k_a ($M^{-1}s^{-1}$)	k_d (s^{-1})	K_D (M)	$T_{1/2}$ (min)	Max RLU	EC_{50} (M)
Full-length anti-IsdB[a]	anti-IsdB x anti-C1q	3.22×10^{6}	5.02×10^{-3}	1.56×10^{-9}	2.3	9.10×10^{5}	1.27×10^{-10}
scFv anti-IsdB	anti-IsdB x anti-C1q scFv	1.23×10^{7}	9.20×10^{-3}	7.48×10^{-10}	1.3	N/D	N/D
IsdA	anti-IsdA x anti-C1q	3.62×10^{6}	4.69×10^{-3}	1.30×10^{-9}	2.5	6.84×10^{5}	1.91×10^{-10}
PNAG	anti-PNAG x anti-C1q	1.61×10^{7}	1.02×10^{-2}	6.32×10^{-10}	1.1	1.33×10^{6}	3.94×10^{-9}
ClfA	anti-ClfA x anti-C1q	4.35×10^{6}	9.20×10^{-3}	7.48×10^{-10}	1.3	1.79×10^{6}	3.21×10^{-8}
Protein A	anti-protein A x anti-C1q	3.60×10^{6}	4.88×10^{-3}	1.36×10^{-9}	2.4	1.24×10^{6}	2.40×10^{-10}

[a]50 nM bispecific antibody was injected across a chip surface of captured His-tagged antigen

[b]90 nM His-tagged IsdB injected across an Fc-captured anti-IsdB chip surface

N/D - Not determined as C1q deposition levels were too low for accurate calculation

Supplementary Table 2 Binding parameters for antibodies binding to their target ligands

Targeting Arm	Capture Surface	Test Ligand	Biacore kinetic parameters for binding at 37 °C				Antibody binding to *S. aureus*	
			k_a ($M^{-1}s^{-1}$)	k_d (s^{-1})	K_D (M)	$T_{1/2}$ (min)	Max RLU	EC_{50} (M)
Full-length anti-IsdB[a]	IsdB.6x(His)	anti-IsdB x anti-C1q	8.74×10^{5}	6.57×10^{-3}	7.52×10^{-9}	1.8	7.93×10^{5}	1.90×10^{-10}
IsdA[a]	IsdA.6x(His)	anti-IsdA x anti-C1q	2.70×10^{5}	9.77×10^{-4}	3.62×10^{-9}	11.8	1.33×10^{6}	8.36×10^{-10}
PNAG	N/A	N/A	N/A	N/A	N/A	N/A	1.62×10^{5}	3.01×10^{-9}
ClfA	N/A	N/A	N/A	N/A	N/A	N/A	3.04×10^{5}	5.69×10^{-10}
Protein A	N/A	N/A	N/A	N/A	N/A	N/A	1.91×10^{5}	2.44×10^{-8}
IsdB[b]	anti-IsdB Bivalent	IsdB.6x(His)	3.65×10^{5}	6.40×10^{-4}	1.75×10^{-9}	18	7.43×10^{5}	1.48×10^{-10}

[a]50 nM bispecific antibody was injected across a chip surface of captured His-tagged antigen

[b]90 nM His-tagged IsdB injected across an Fc-captured anti-IsdB chip surface

Supplementary Table 3 Relative levels of *S. aureus* surface antigens

Surface Antigen	Copy number relative to isotype control
IsdB[a]	7.26
ClfA[a]	0.90
PNAG[a]	0.71
IsdA[b]	13.10

[a]Experiment was performed twice using an anti-Fel D1 antibody as the isotype control
[b]Experiment was performed once using an anti-CD28 antibody as the isotype control

Supplementary Figure 7

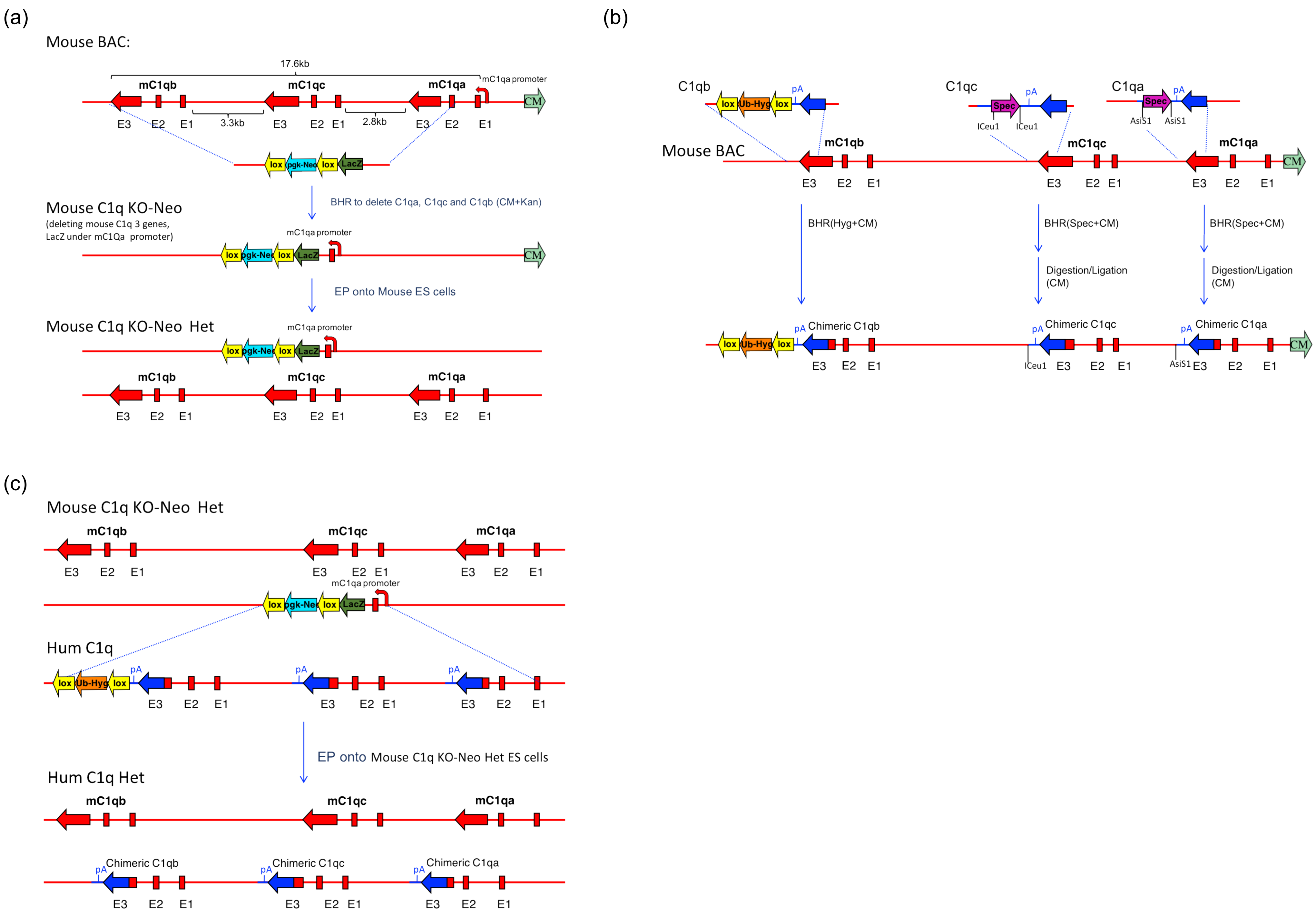

Supplementary Table 4 Primers for cloning BACs used in C1q humanized mouse production

Mouse	Primer name	Sequence
Mouse C1q KO	SU	CATTGTTAGTGCTAGTATCGG
	HUF	GACCTGTGATCCAGACAGG
	BUR	ACACTGGGTATGCTGGACAC
	HUR	CATGGTGTCCCTACAGAGAC
	BDF	TGCCAACAGCATCTTCACTG
	HDF	CTATCCAACACCATCTTCCTG
	HDR	GACTCAGACTCAGATTAGACC
	SD	GCCTGAGAGGTGTTAGTCAA
Humanization C1q	5'up detect (C1Qa-m65)	ATGGCTGCATTTGCTTCTCT
	5'upC1Qa(m51a)	CTGTGAGGATAGGCAGGCTC
	3'up-C1Qa-M-H(m50)	GGCTGGCCTCGGCTGGTCCCTGATATTGCCTGGATTG
	3'up detect (C1Qa-m66)	ACGATGGACAGGCAGATTTC
	3'up detect (C1Qa-m66)	ACGATGGACAGGCAGATTTC
	5'down detect (C1Qa-m68)	TTCCTCATCTTCCCATCTGC
	5'down (m52)	TGATGCACGCCTTTAATCCCAGC
	3'spec-p159-AsisI-DB	TAAAGGCGTGCATCAGCGATCGC
	3' C1Qa-M-SalI-pUni(m53)	GGCACCACTTAGCAGATTTGG
	3'down detect (C1Qa-m67)	GACTGGGAGGGTATTGGTCA
	5'up detect (C1Qc-m69)	CTCCCACCTCAGATTCCAAA
	5'up-C1Qc-pUni-SalI(m55)	CTGTCCCCTCCACCTGGGCAG
	3'up-C1Qc-M-H(m56)	GAATTTCTGCTTGTATCGGCCCTCCACACCTGGCTC
	5'spec-p159(c)	TACCTAACTATAACGGTCC
	3' C1Qc-M-SalI-pUni(m58)	GCCTCACAGCTTGTACAAACAG
	3'down detect (C1Qc-m71)	CTCTTTCATACCGCTCTGCC
	5'up detect (C1Qb-m72)	GAAGCCCTGCGTATCAGTTC
	3'up-C1Qb-M-H(m60)	GAAGGCGATTTTCTGTGTAGCCCCGTAGTCCCCAG
	3'C1Qb-H-lox-HYG(h61a)	CCGCATTCATTCATGTAAC
	3' ub 200	CCAGTGCCCTAGAGTCACCCA
	5'down(m62a)	ACGAAGTTATGTCGACGAATGTTCATAGGCTGGGG
	3' C1Qb-M-SalI-pUni(m63)	GGTCCAGAGAGAACCACAATG
	3'down detect (C1Qb-m86)	CATTGTGGTTCTCTCTGGACC
	3'down detect (C1Qb-m74)	CAGATCATCATGGAGGGCTT

Supplementary Table 5 qPCR probes and primers used for screening mouse stem cells

Probe name	Forward primer	Probe	Reverse primer
596TUP	TAAGCGTTCTCTCCGGCTGG	TCCCGCACCATCCTGGAGGCAAT	CGCTTCTCAGGACCCCTAAAC
Neo probe	GGTGGAGAGGCTATTCGGC	TGGGCACAACAGACAATCGGCTG	GAACACGGCGGCATCAG
LacZ probe	GGAGTGCGATCTTCCTGAGG	CGATACTGTCGTCGTCCCCTCAAACTG	CGCATCGTAACCGTGCATC
597D Probe	GAAAGTCGCCTTCTCTGCCC	AGGACCATCAACAGCCCCTTGCGAC	CGAAGCGAATGACCTGGTTC
1565ma1	CGCTTGGCAACGTGGTTAT	TGACAAGGTCCTCACCAACCAGGAGAG	CCCGTGTGGTTCTGGTATGG
1565mb1	TCACCAACGCGAACGAGAA	TATGAGCCACGCAACGGCAAGTTCA	GGCCAGGCACCTTGCA
1565mc5	CACCTCGCTCCCTCTGCTT	CCCATCCTCACTCAGACCTCTTCCTCCA	CAGGAACCAGGGTGGACTTC
1565ha1	CGGAACCCCCCAATGG	CAACGTGGTCATCTTCGACACGGTCA	TGGTTCTGGTACGGTTCTTCCT
1565hb2	AATCGCCTTCTCTGCCACAA	ACCATCAACGTCCCCCTGCGC	GTGGTCGAAGCGGATGGT
1565hc4	TGACACGAGCACTGGCAAGT	CACCTGCAAAGTCCCCGGCCTC	CGACGCGTGGTAGACAAAGTAG
Hyg probe	TGCGGCCGATCTTAGCC	ACGAGCGGGTTCGGCCCATTC	TTGACCGATTCCTTGCGG

Supplementary Figure 8

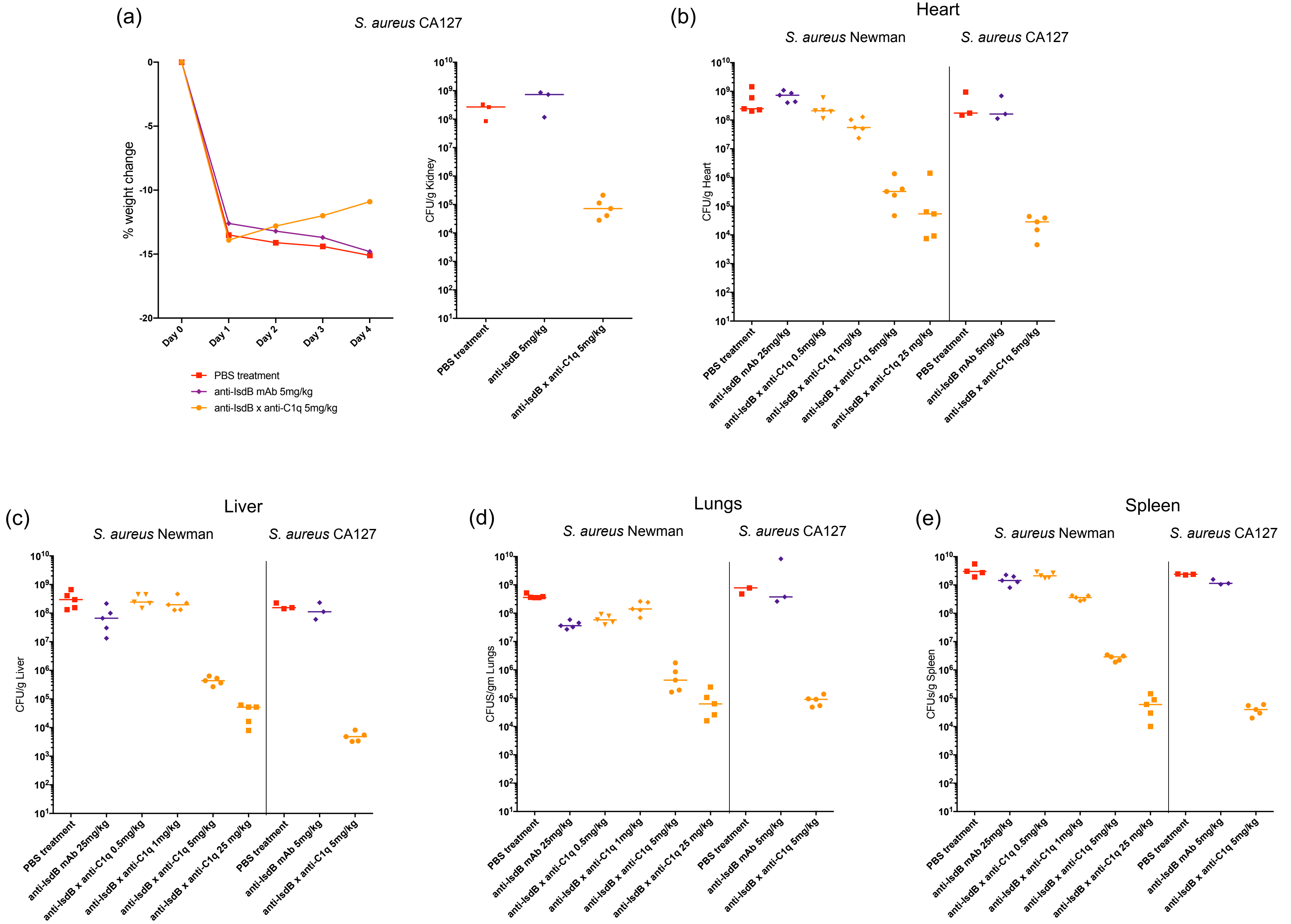

Supplementary Table 6 Binding parameters for 5 nM C1q binding as the test ligand to antibody captured chip surfaces

		Biacore kinetic parameters for binding at 37 ^{o}C			
**Targeting Arm**	**Capture Surface**	**k_a ($M^{-1}s^{-1}$)**	**k_d (s^{-1})**	**K_D (M)**	**$T_{1/2}$ (min)**
Psl	anti-Psl x anti-C1q	4.80×10^{6}	1.16×10^{-2}	2.41×10^{-9}	1
CD20	anti-CD20 x anti-C1q	1.04×10^{7}	4.99×10^{-3}	4.81×10^{-10}	2.3
GITR	anti-GITR-anti-C1q	3.34×10^{6}	5.82×10^{-3}	1.74×10^{-9}	2

Supplementary Table 7 Binding parameters for bispecific antibody binding to ligand captured chip surfaces

Targeting Arm	Capture Surface	Test Ligand (50 nM)	Biacore kinetic parameters for binding at 37 °C			
			k_a ($M^{-1}s^{-1}$)	k_d (s^{-1})	K_D (M)	$T_{1/2}$ (min)
GITR	hGITR.mmh	anti-GITR x anti-C1q	1.19×10^{6}	1.90×10^{-2}	1.60×10^{-8}	0.6

Supplementary Figure 9

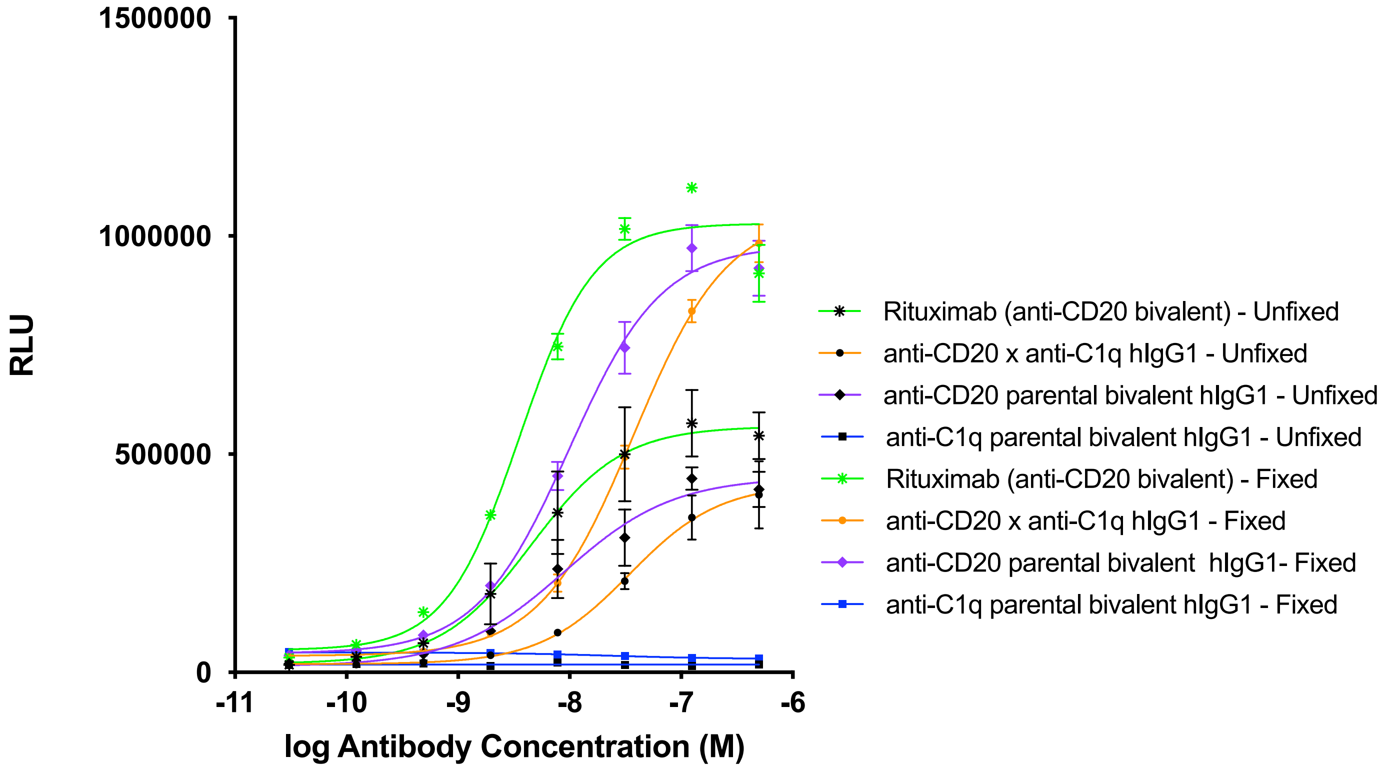

Antibody EC_{50} Values (M)

Unfix/Fix	Rituximab (anti-CD20 bivalent)	anti-CD20 x anti-C1q hIgG1	anti-CD20 parental bivalent hIgG1	anti-C1q hIgG1 parental bivalent
Unfixed	4.52×10^{-9}	3.45×10^{-8}	8.96×10^{-9}	No Binding
Fixed	3.56×10^{-9}	3.75×10^{-8}	9.88×10^{-9}	No Binding

Supplementary Figure 10

(a)

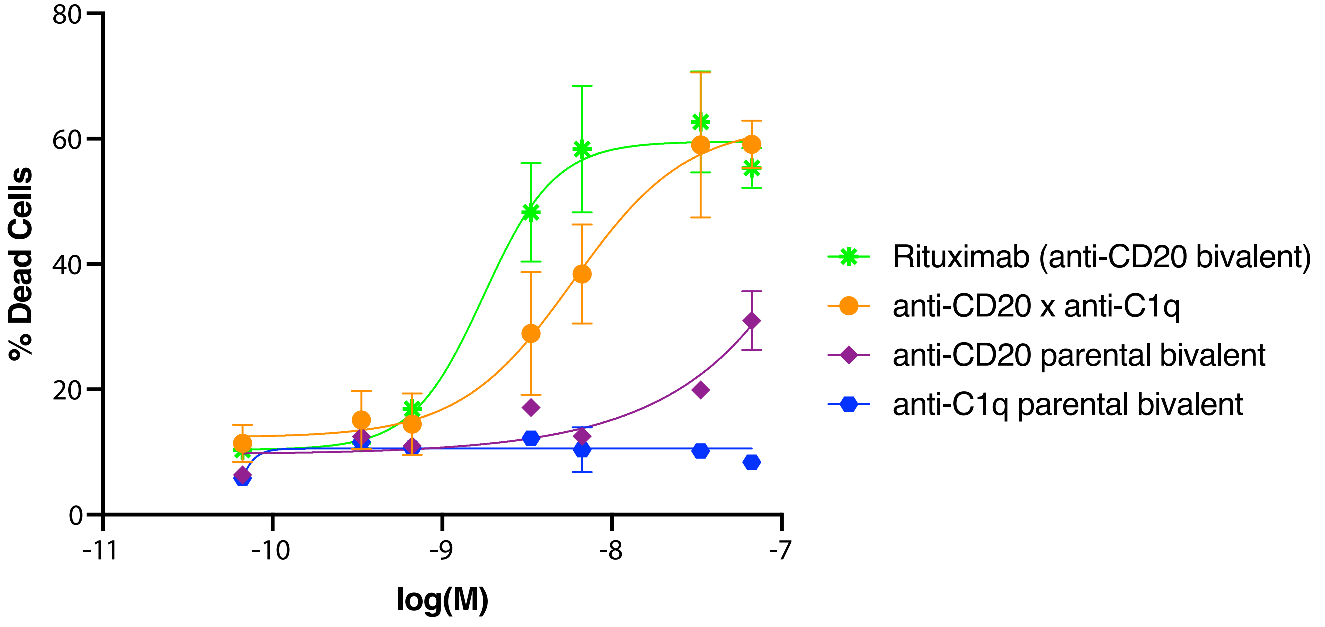

Antibody	Cell type	EC_{50} 5% Serum(M)	EC_{50} 50% Serum(M)
anti-CD20 x anti-C1q bsAb	Raji	$2.04x10^{-9}$	$5.92x10^{-9}$
Rituximab, anti-CD20 bivalent	Raji	$6.32x10^{-10}$	$1.76x10^{-9}$
anti-CD20 parental bivalent	Raji	NC	NC
anti-C1q parental bivalent	Raji	NC	NC

(b)

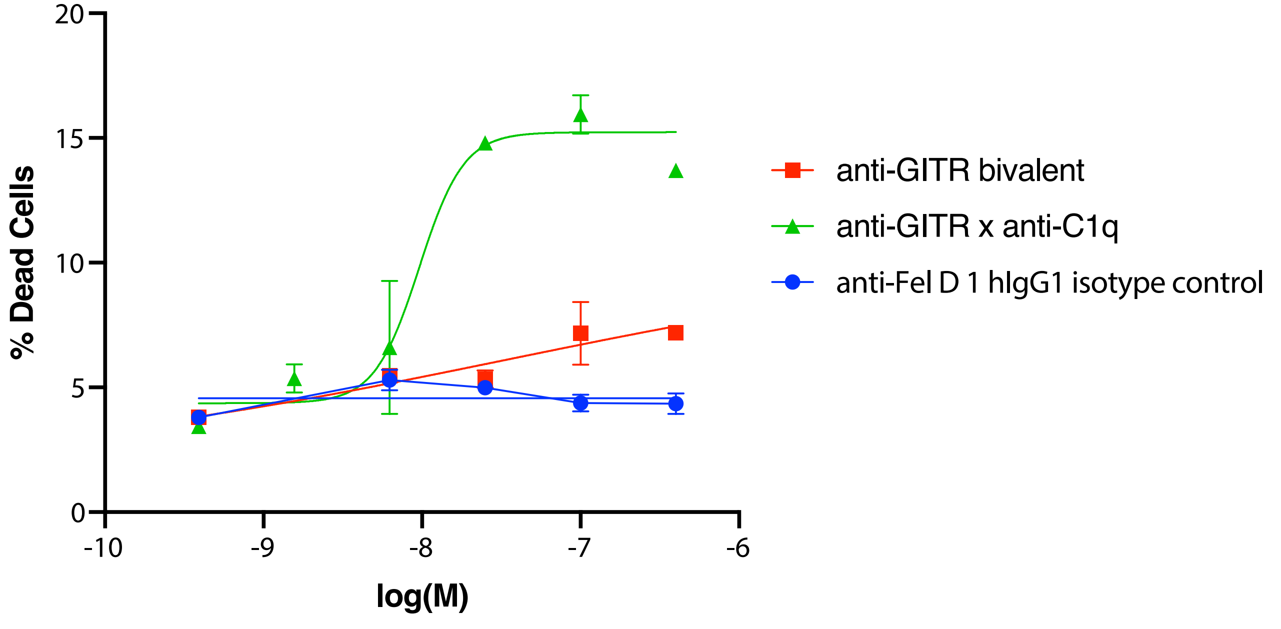

Antibody	Cell type	EC_{50} 5% Serum(M)	EC_{50} 50% Serum(M)
anti-GITR x anti-C1q bsAb	Jurkat/hGITR/hDC20	$1.97x10^{-8}$	$9.56x10^{-9}$
anti-GITR bivalent	Jurkat/hGITR/hDC20	NC	NC
anti-FelD1 isotype control	Jurkat/hGITR/hDC20	NC	NC

Supplementary Table 8 Relative level of complement inhibitors on tested cell types

Cell Line	CD55 fold change compared to isotype control	CD59 fold change compared to isotype control
Raji (B-cell line)	7.7	6.2
Jurkat (T-cell line)	23.4	53.1
